# Synthesis of a Luminescent Aluminum-Based MOF for Selective Iron(III) Ion Sensing

**DOI:** 10.3390/molecules30204146

**Published:** 2025-10-21

**Authors:** Hanibal Othman, István Boldog, Christoph Janiak

**Affiliations:** Institut für Anorganische Chemie und Strukturchemie, Heinrich-Heine-Universität Düsseldorf, D-40204 Dusseldorf, Germany; hanibal.othman@hhu.de (H.O.); Istvan.Boldog@hhu.de (I.B.)

**Keywords:** photoluminescence, metal–organic framework, limit of detection, iron sensing, Stern-Volmer equation

## Abstract

In the search for new materials to open up creative pathways for industry and research, modification is one of the best methods to implement. Developing materials with high sensitivity and selectivity for specific applications, such as ion sensing, remains a significant challenge. This work aims to introduce a novel metal–organic framework (MOF) derived from the well-established 2-amino-[1,1′-biphenyl]-4,4′-dicarboxylic acid MOF by modifying its structure to enhance its properties and applications. A luminescent 2-naphthyl moiety was attached to the amino group of the linker to form the new luminescent Al-based MOF Al-BP-Naph with a surface area of 456 m^2^ g^−1^ and a pore volume of 0.55 cm^3^ g^−1^. Al-BP-Naph showed high selectivity towards Fe^3+^ sensing due to the overlapping absorption and excitation spectra of both Fe^3+^ and MOF. The MOF demonstrated a detection limit of approximately 6 × 10^−6^ mol L^−1^ with a limit of quantification of about 19 × 10^−6^ mol L^−1^ and a very fast response time (less than 10 s). It also had a Stern–Volmer constant of approximately 0.09 × 10^5^ L mol^−1^, distinguishing it from other ions. Our work contributes to the expanding repertoire of functional materials with promising applications in sensing technologies, offering a novel MOF with superior properties for iron(III) ion detection.

## 1. Introduction

The development of sensors and materials that respond selectively to specific analytes is progressing for improved sensitivity, selectivity, and the range of detectable substances. These advancements are being driven by the need for more precise, reliable, and efficient analyte detection systems across various scientific and industrial fields. A particularly important area within the realm of sensing is photoluminescence sensing. The photoluminescent response can be classified as either positive or negative. In a positive response, known as photoluminescence enhancement or a “turn-on” reaction, the material’s light emission increases when it encounters the analyte [1,2,3,4]. Conversely, in a negative response, referred to as photoluminescence quenching or a “turn-off” reaction, the material’s emission is diminished or entirely suppressed in the presence of the analyte [5]. This dual capability makes photoluminescent sensors highly versatile and valuable in a wide range of applications, from environmental monitoring to biomedical diagnostics [6,7,8,9,10].

Iron is a critical element in both biological and industrial contexts and exemplifies the importance of precise analyte detection. In biological systems, iron plays a vital role in various physiological processes, including oxygen transport, DNA synthesis, and electron transport within cells [11]. However, both iron deficiency and iron overload can lead to severe health complications, such as anemia in the case of deficiency or tissue damage and organ failure in cases of iron overload. Therefore, accurate quantification of iron in biological samples is crucial for effective medical diagnosis and treatment [12,13].

From industrial processes, large quantities of iron and other metals can be released into rivers, soil, and air, posing serious environmental and health risks. This necessitates stringent regulatory measures to monitor and limit the concentration of iron and other potentially harmful metals in the environment [14].

**Scheme 1:**
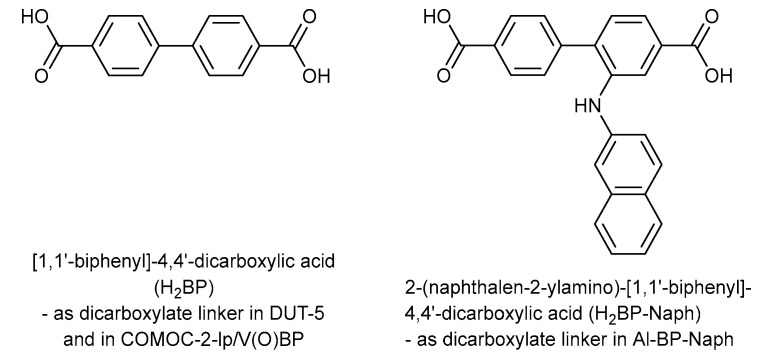
Relevant linkers for the MOF structures in this work.

Metal–organic frameworks (MOFs) are emerging as powerful tools in the field of sensing. MOFs are porous structures formed through the coordination of metal ions with bridging organic linkers [15]. MOFs possess highly tunable properties, making them suitable for a wide array of applications, including photoluminescence sensing [16,17,18,19]. As photoluminescent sensors, MOFs have demonstrated exceptional sensitivity, capable of detecting a wide range of metal ions [20,21,22]. In this study, we introduce an aluminum metal–organic framework related to DUT-5 (Al(OH)(1,1′-biphenyl]-4,4′-dicarboxylate) [23], but synthesized using 2-(naphthalen-2-ylamino)-[1,1′-biphenyl]-4,4′-dicarboxylic acid (H_2_BP-Naph), where we added the naphthylamino group to the biphenyl linker, achieving a strongly luminescent MOF (Scheme 1) [24]. The napthalene-2-ylamino luminophore group, and hence this newly developed MOF Al-BP-Naph, exhibits photoluminescent properties and experiences luminescence quenching in the presence of metal ions, most notably Fe^3+^. We have investigated the quenching selectivity and concentration dependence for a potential sensing application of Al-BP-Naph. In line with related investigations in the literature, the MOF was applied as an N,N-dimethylformamide (DMF) dispersion because of the DMF properties with regard to polarity for metal-salt solubility and transparency in the UV-Vis region, due to its high refractive index (1.43), which minimizes the mismatch between the optical medium (quartz cuvettes) and the sample [25].

This work introduces Al-BP-Naph, a novel Al-based MOF that combines the stability of MIL-53-type frameworks with a naphthylamino-functionalized linker acting as an intrinsic luminophore. This MOF enables highly selective and rapid quenching by Fe^3+^ through spectral overlap, giving a low detection limit (5.6 µmol L^−1^) and sub-10 s response time. These properties surpass many reported MOF sensors, highlighting the distinctiveness and novelty of Al-BP-Naph in luminescent iron sensing.

## 2. Results

**Figure 1:**
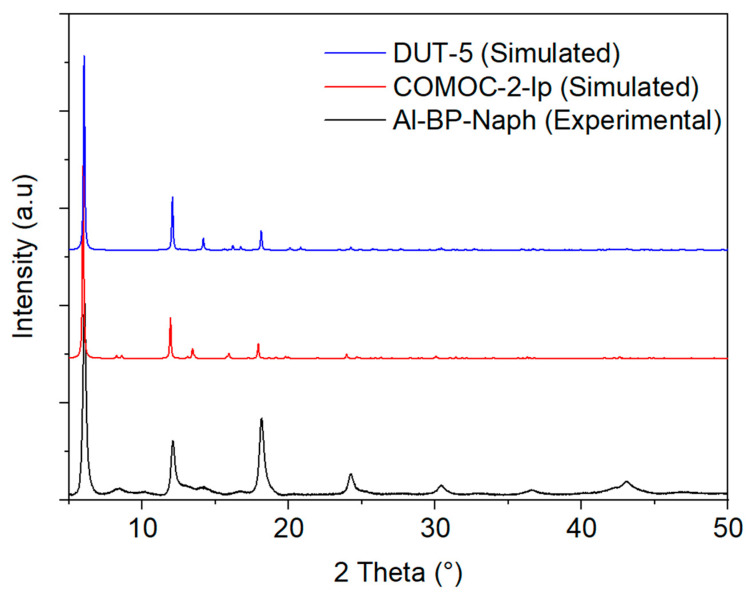
PXRD comparison between Al-BP-Naph dried at 70 °C for 24 h using a vacuum oven (1 × 10^−3^ bar), and the simulation for DUT-5 and COMOC-2-lp from the CCDC ref codes 691979 and 868306, respectively [23,27]. The background of the experimental pattern was removed (see the uncorrected pattern in Figure S4).

The MOF compound Al-BP-Naph was synthesized from 2-(naphthalen-2-ylamino)-[1,1′-biphenyl]-4,4′-dicarboxylic acid [26] and aluminum nitrate nonahydrate in DMF. The structural analogy of the synthesized material Al-BP-Naph to the MOFs DUT-5 [23] and the related COMOC-2-lp/V(O)BP [27] was confirmed by powder X-ray powder diffraction (PXRD) studies (Figure 1). The material is generally crystalline with a well-defined short-to-medium-range periodicity; however, there are also indications of compromised crystallinity, witnessed by a relatively high background. It is worth noting in this context that aluminum MOFs tend to form products with at least partially compromised crystallinity due to the low reversibility of the crystallization process. This is also the reason why Al-MOFs are often hard to obtain in single crystalline form for diffraction studies, which is also the case here.

**Figure 2:**
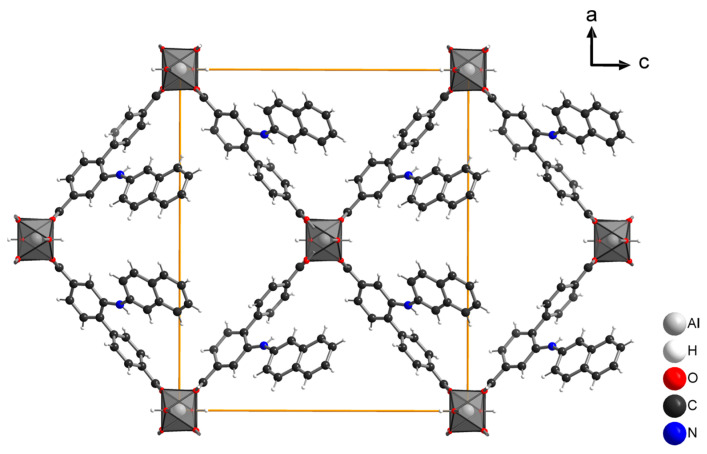
View along the b-axis for the structure of Al-BP-Naph with the “wine-rack” MIL-53 topology, demonstrating an approximate possible regular localization of the pendant naphthylamino group. The orange line is the unit cell outline.

The indicators of somewhat lowered crystallinity are also expected as manifestations of structural flexibility. The latter is inherent in the “wine-rack” type MIL-53 structure (Figure 2), which can potentially adapt a continuous range of geometries within the Imma space group, which was assumed to be the same as in the case of COMOC-2-lp/V(O)BPDC and DUT-5 (see Figures S4–S9 and Table S1) [23,27].

The cell parameters vary slightly for the three compounds, which represent the open form of the MIL-53-type structure. Despite the same length of the ligand, the discussed slight flexibility of the framework, associated with the hinge-like movement of the ligands related to the secondary building unit (see Figure S6 for the cell overlap of Al-BP-Naph and COMOC-2-lp), can lead to a difference in the length of the a and c axis (cf. Figure 2; elongation of c when a becomes shorter and vice versa).

The low crystallinity can be further attributed to the disorder and amorphosity introduced into the lattice by the naphthylamino group attached to the side of the linker. The presence of this pendant group creates significant variability in the crystal structure because its orientation within the pore framework can occur on both sides of the linker (Figure 2). The significant disorder in this part of the structure restricted the refinement to the Le Bail method, as described in the Supplementary Materials [27].

Furthermore, the stability of the Al-BP-Naph material after being exposed to ambient air conditions at RT for 7 days and after activation for adsorption measurements was positively verified by PXRD (Figure S9), in line with the usual high stability of the MIL-53(Al)-type materials. Al-BP-Naph retained its crystallinity under these conditions, which is important for practical applications where prolonged exposure to ambient conditions is expected. However, its structural integrity was largely lost when heated to 90 °C for 24 h (Figure S9).

Thermogravimetric analysis (TGA) in Figure S11 of the dried MOF with the molecular formula [Al(OH)(BP-Naph)] = (AlC_24_H_16_NO_5_) (revealed an initial mass loss of 10% between 60 °C and 160 °C, which is attributed to the release of residual ethanol and DMF solvents used during synthesis and trapped within the porous structure. Following this mass loss, a plateau is observed until approximately 400–500 °C, where a significant mass loss of 77% occurs, corresponding to the thermal decomposition of the organic linker (BP-Naph). When the contribution of the solvent loss is excluded and the mass at 225 °C is rescaled to 100%, the mass loss around 1000 °C becomes 88%, which matches the theoretical linker content (89.7%) of C_24_H_15_NO_4_ in AlC_24_H_16_NO_5_. The corrected residual mass at 1000 °C becomes 11.7% which agrees with the expected amount of Al_2_O_3_ (theor. 11.98% for 6.34 wt% of Al). Since the analysis was conducted under a synthetic air atmosphere, it is reasonable to assume that all aluminum present in the framework was fully converted to Al_2_O_3_.

IR spectroscopy in Figure S10 shows a shift in the C=O stretching mode between the linker and the MOF, indicating the coordination of the carboxylate group to the metal and also demonstrating the near absence of the non-deprotonated linker pore-filling impurity, which is typical for such compounds.

**Figure 3:**
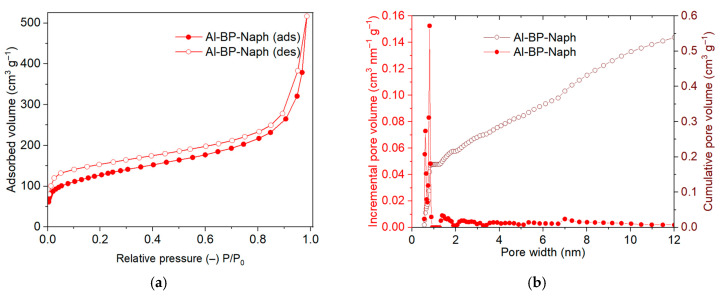
(**a**) Al-BP-Naph N_2_ sorption isotherm at 77 K (filled symbols: adsorption, empty symbols: desorption). (**b**) Pore size distribution using the slit pore, NLDFT equilibrium model.

The surface area and porosity of Al-BP-Naph were analyzed through N_2_ adsorption studies at 77 K. The adsorption isotherm of Al-BP-Naph is a combination of type I at lower and type II at higher relative pressures (Figure 3a). The steep uptake at low P/P_0_ is indicative of a microporous material giving the type I isotherm. Type II isotherms stem from the physisorption on nonporous or macroporous adsorbents, here from the interparticle condensation. The composite type I and II isotherms often have an H4 hysteresis loop, which is also observed with aggregated crystals of zeolites, some mesoporous zeolites, and micro-mesoporous carbons [28]. From the adsorption branch, a BET surface area of approximately 456 m^2^ g^−1^ was calculated (over a pressure range of P/P_0_ = 0.04–0.1 with a correlation coefficient R^2^ of 0.999 and a BET constant C of 183.9, Figure S13). The total pore volume was found to be 0.546 cm^3^ g^–1^, with a micropore volume of 0.215 cm^3^ g^−1^. For DUT-5, the surface area is 1413–1467 m^2^ g^–1^, and the pore volume is ~0.71 cm^3^ g^−1^ [29,30]. The pore size distribution from NLDFT calculations indicates a fraction of 40% of micropores (<2 nm) and 60% mesopores (2–50 nm) (Figure 3b).

**Figure 4:**
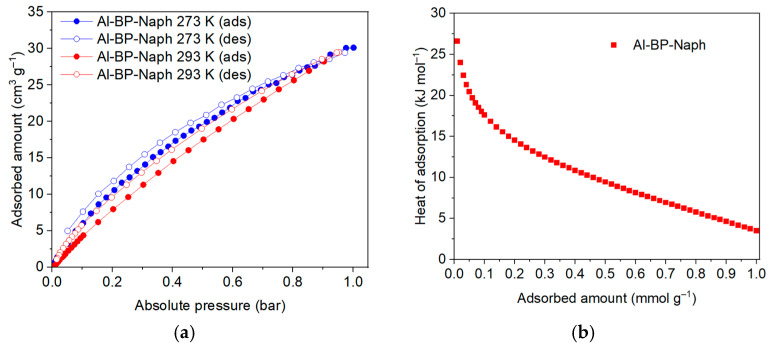
(**a**) CO_2_ sorption isotherms of Al-BP-Naph at 273 K and 293 K (filled symbols: adsorption, empty symbols: desorption). (**b**) Isosteric heat of CO_2_ adsorption in Al-BP-Naph with increasing uptake.

CO_2_ adsorption of Al-BP-Naph was examined at 273 K and 293 K with uptakes of 30.1 cm^3^ g^−1^ and 29.4 cm^3^ g^−1^, respectively. This CO_2_ uptake is similar to the biphenyl-based MOFs of the same structure, such as DUT-5 (36.2 cm^3^ g^−1^ at 298 K) [29] and COMOC-2 (19.0 cm^3^ g^−1^) [31]. From the uptake at the two temperatures, the isosteric heat of adsorption near zero coverage was computed to 26 kJ mol^–1^ (Figure 4b), and this value drops with increasing uptake at 1 mmol g^–1^ to about 3.5 kJ mol^–1^, which is below the heat of liquefaction of CO_2_ [32] (see Section S6 for details). The heat of adsorption or the affinity for CO_2_ diminishes with increasing uptake, as the higher-binding energy sites become increasingly occupied [33,34]. This isosteric heat of adsorption near zero coverage for CO_2_ is similar to the values of UiO-67-(NH_2_)_2_- [35] and COMOC-2 [31] with around 30 kJ mol^−1^ and to other MOFs with a biphenyl linker (Table S3) [36,37,38].

**Figure 5:**
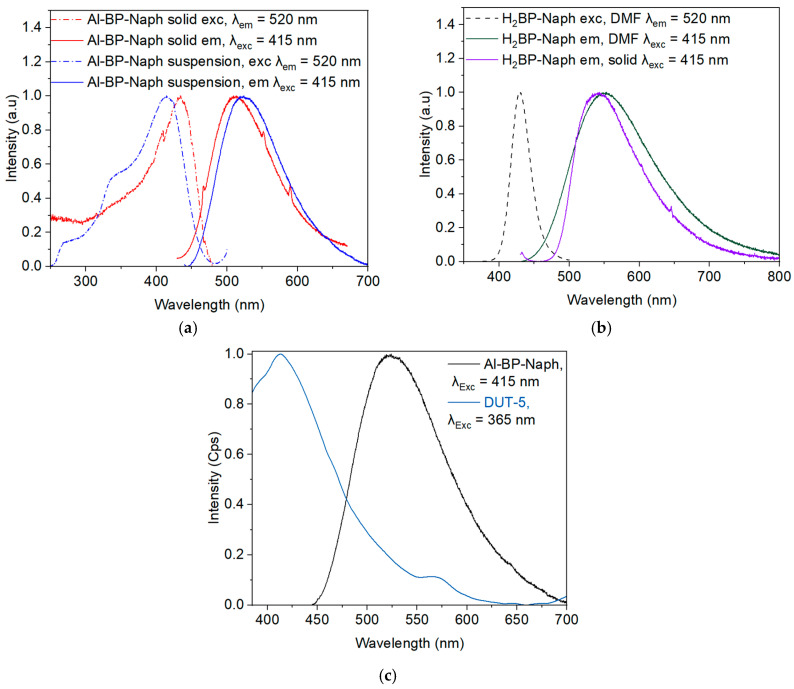
(**a**) Excitation (dashed lines) and steady-state photoluminescence spectra (solid lines) of Al-BP-Naph in solid form (red) and suspended (blue curves) in DMF. (**b**) H_2_BP-Naph linker excitation and emission spectrum in DMF and the solid state. (**c**) Emission spectra of Al-BP-Naph and DUT-5, where Al-BP-Naph was excited at λ_exc_ = 415 nm and DUT-5 at λ_exc_ = 365 nm.

The newly synthesized Al-based metal–organic framework (Al-BP-Naph) incorporates a naphthyl-substituted biphenyl linker bearing a naphthylamino functionality, which acts as a separate luminophore and enhances the framework’s luminescence. Al-BP-Naph exhibits intrinsic photoluminescence, with an emission maximum observed in DMF suspension at 520 nm (Figure 5). A slight solvatochromic shift is noted compared to the emission of the dry solid-state (510 nm), which is attributed to interactions between the naphthyl group in the MOF and the adsorbed solvent molecules that modulate the electronic environment and optical behavior, as it is also seen for the free linker H_2_PB-Naph when comparing the solid-state and its DMF solution (Figure 5b).

**Figure 6:**
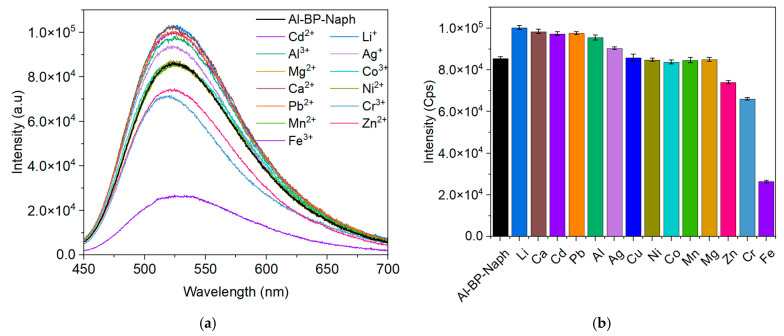
(**a**) Emission spectra (λ_exc_ = 415 nm) of Al-BP-Naph (as DMF suspension of 1 g L^–1^) in the presence of different metal ions at concentrations of 0.003 mol L^–1^. (**b**) Luminescence intensity comparison of Al-BP-Naph for different metal ions with concentrations and ion charges as in (**a**). The intensity error was estimated to ±0.2 × 10^4^.

The emission of Al-BP-Naph was tested for the presence of metal ions in the DMF dispersion of the MOF and found to lead to various degrees of quenching of the emission intensity at 520 nm when the suspension was excited with 415 nm light. Fe^3+^ ions (c = 0.001 mol L^–1^, added as Fe(NO_3_)_3_) result in strong emission quenching by about 75%. The metal ions Cr^3+^ and Zn^2+^ are quenched by only 22% and 13%, respectively, whereas the metal ions Mg^2+^, Mn^2+^, Co^3+^, and Ni^2+^ leave the emission intensity unchanged, and, in contrast, the ions Ag^+^, Al^3+^, Pb^2+^, Cd^2+^, Ca^2+^ and Li^+^ even enhance the luminescence by about 10–20% (all at a concentration of 0.1 mol L^–1^) (Figure 6).

**Figure 7:**
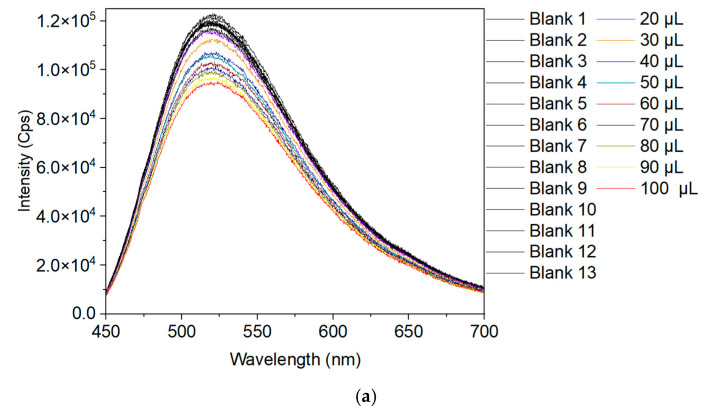

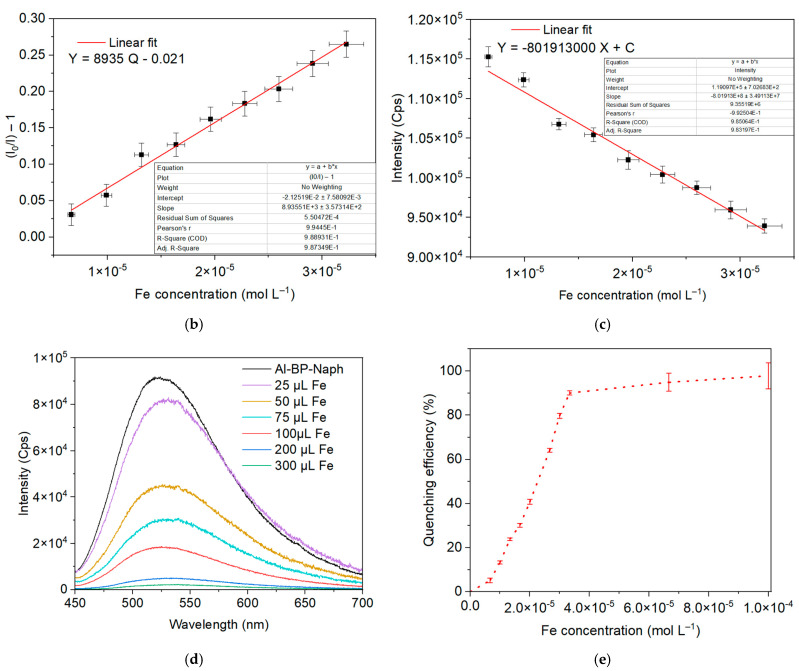
(**a**) Al-BP-Naph emission response (λ_exc_ = 415 nm) in DMF suspension (3.0 mL) to increasingly added volumes of a DMF Fe^3+^ solution (c = 0.001 mol L^–1^) (thirteen measurements for the blank sample Al-BP-Naph), (**b**) Stern–Volmer plot for the fluorescence intensity of Al-BP-Naph to different concentrations of Fe^3+^, (**c**) fit of the fluorescence intensity versus analyte ion concentration, (**d**) Al-BP-Naph response as DMF suspension to different amounts/concentrations of Fe^3+^ for the calculation of the quenching efficiency, (**e**) quenching efficiency to concentrations of Fe^3+^. In (**b**) the error σ for (I_0_/I) − 1 was derived from the general law of propagation of uncertainty σ = (I_0_/I)r √ 2 and r = 1% from the relative error in the emission intensities. In (**c**) the intensity error was estimated to be ±0.2 × 10^4^. The accuracy of the Fe^3+^ concentration was estimated with its dissolution and dilution procedures to ±5% and the error bars were added accordingly to (**b**,**c**) for the Fe-concentration axis. The error of the quenching efficiency in (**e**) was derived by calculating the relative error at each absorption maximum in (**d**) from the noise, divided by the absorption intensity, and adding this value as the error bar to the efficiency axis.

As it was found that the emission of Al-BP-Naph is particularly quenched by Fe^3+^ ions (Figure 6), an increase in Fe^3+^ concentration led to a progressive quenching of the emission and was quantitatively analyzed using the Stern–Volmer Equation (1), which revealed a relatively high Stern-Volmer constant of 0.09 × 10^5^ L mol^–1^ (Figure 7b). This high constant indicates that Fe^3+^ is an effective quencher of the luminescence of Al-BP-Naph.

I_0_/I − 1 = K_SV_ Q (1)

where

I_0_: Luminescence intensity of pristine Al-BP-Naph in DMF suspension.
I: Luminescence intensity of Al-BP-Naph in the presence of Fe(III) ions in DMF
K_SV_: Stern-Volmer constant
Q: Concentration of the quenching Fe^3+^ ions

The proposed mechanism for the observed quenching effect is suggested to involve competitive absorption of the energy required for the excitation of the MOF. In this scenario, the energy intended to excite the MOF is instead absorbed by another species—in this case, the cation from the metal nitrate that is in direct contact with the MOF structure. The quenching effect observed in the presence of iron ions can be attributed to the overlap between the absorption spectrum of Fe^3+^ ions, which typically spans the wavelength range of 200 to 450 nm [34], and the excitation spectrum of Al-BP-Naph in DMF suspension (Figure S15). This spectral overlap results in a significant portion of the excitation energy being absorbed by the iron ions rather than by the MOF itself. Consequently, this absorption by Fe^3+^ ions leads to a reduction in the energy available for exciting the MOF, which in turn causes a pronounced quenching of the emission in the suspension. In contrast, the absorption spectra of other metal nitrates, which were also tested, showed less overlap with the MOF excitation region (Figure S15), suggesting a reduced ability to interfere with the MOF’s excitation process. These findings lend further credence to the idea that iron nitrate uniquely quenches the MOF’s fluorescence via this competitive absorption mechanism.

We dismiss static quenching, that is, a complex formation between the naphthylamino fluorophore and Fe^3+^, because among the tested metal ions, also Cr^3+^, Mn^2+^, Co^2+^, Ni^2+^, Cu^2+^, and Cd^2+^ are known to readily form amine complexes. Dynamic quenching can occur when a quencher molecule collides with the excited fluorophore, transferring energy or electrons and returning the fluorophore to its ground state non-radiatively, which decreases fluorescence intensity but can also be excluded as the radius of high-spin Fe^3+^ is 0.78 Å, which is larger than for Al^3+^, Co^2+^ and Cr^3+^ and smaller than for Mg^2+^, Li^+^, Pb^2+^, Ni^2+^, Mn^2+^, Cd^2+^, Ag^+^, Ca^2+^, Zn^2+^ and Cu^2+^ [39]. Thus, at least the ions Al^3+^, Co^2+^ and Cr^3+^ (which are smaller than Fe^3+^) could diffuse into the MOF similar to Fe^3+^ and result in dynamic quenching, which is, however, not seen.

The luminescence intensity at 520 nm displayed a negative linear correlation with Fe^3+^ concentration within the range of 6.6 × 10^–6^ to 3.2 × 10^–5^ mol L^–1^, with a correlation coefficient (R^2^) greater than 0.98, as illustrated in Figure 7b,c. This strong linear relationship allows for precise quantification of Fe^3+^ concentrations. The Stern-Volmer quenching constant (K_SV_) calculated from these measurements reached a value of 0.09 × 10^5^ L mol^−1^, which underscores the high efficiency of iron ions in quenching the luminescence of the MOF. The quenching efficiency was calculated using the formula (I_0_/I − 1) × 100% where I_0_ and I are the values of the luminescence intensity of pristine Al-BP-Naph- and Fe^3+^-loaded Al-BP-Naph, respectively, indicating that Al-BP-Naph showed a high sensitivity in sensing fluorescence quenching. The quenching efficiency was close to 100% when the added volume of the Fe^3+^ solution reached 300 µL (giving an Fe^3+^ concentration of 10^–4^ mol L^–1^ in the Al-BP-Naph suspension) (Figure 7d,e).

Additionally, the limit of detection (LOD) for iron(III) nitrate was determined as low as about 6 µmol L^–1^. This was calculated using the equation LOD = 3 σ/k, where σ represents the standard deviation of the 13 blank measurements (Table S4) and k denotes the slope of the fitting line of fluorescence intensity versus analyte ion concentration in Figure 7c. This low detection limit demonstrates the exceptional sensitivity of Al-BP-Naph and its capability for detecting very small concentrations of iron ions in solution. The limit of detection of Al-BP-Naph is lower than in many other MOFs in the field of iron detection, such as CdBPTC, Ni-MOF, Zn-MOF, and Cd-MOF with LODs of 17, 15, 28, and 57 µmol L^–1^, respectively [16,40,41]. A detailed comparison can be seen in Table 1. The limit of quantification was also calculated with the equation LOQ = 10 σ/k with a value of 19 µmol L^–1^. The MOF Al-BP-Naph presented in this work shows one of the best results when it comes to the limit of detection for Fe^3+^.

The slight luminescence enhancement that is observed with Ag^+^, Al^3+^, Pb^2+^, Cd^2+^, Ca^2+^ and Li^+^ (Figure 6a) can be a result of the adsorption of these cations on the surface of Al-BP-Naph, perhaps through interaction between naphthyl-π system and the cations causing a slight change in the electronic properties of the naphthyl moiety, enhancing in turn the radiative relaxation pathways [42,43,44].

**Table 1:** Literature comparison for MOFs as Fe^3+^ sensors.

Sample	Solvent	LOD [µmol L^–1^] ^(a)^	LOD [µg mL^–1^] ^(a)^	K_SV_ [L mol^–1^] ^(b)^	Ref.
This work	DMF	5.6	0.31	0.09 × 10^5^	This work
Cu_0.1_/Zn-MOF	Water	833	46.51	0.27 × 10^5^	[12]
EuBDC-OMe	Water	2.9	0.16	0.18 × 10^5^	[13]
CdBPTC	Water	16.6	0.92	0.08 × 10^5^	[17]
[Eu(BTPCA)(H_2_O)]	DMF	10	0.55	-	[20]
Ni-MOF	EtOH	15	0.86	-	[40]
DMAC	DMAC	60	3.35	0.78 × 10^–5^	[45]
EuOHBDC	Water	1.17	0.06	45.64	[46]
NNU-1	Water	200	11.2	-	[47]
Zn-MOF	Water	28	1.56	0.16 × 10^5^	[41]
Cd-MOF	Water	57	3.18	0.10 × 10^5^	[41]
[Eu(H_2_O)_2_(BTMIPA)]	Water	10	0.55	-	[48]
[Zn_2_(L)_2_(bpe)_2_(H2O)_2_]	Water	25	1.39		[49]
MI-53-(Al)	Water	0.9	0.05	-	[50]
[Tb(HL)(DMF)(H_2_O)_2_] 3H_2_O	Water	50	2.79	0.04 × 10^5^	[51]
CALIX@UiO-66-NH_2_	Water	6.7	0.37	0.02 × 10^5^	[52]
Eu(L1)_3_	Water	100	5.58	-	[53]
FJI-C8	DMF	23	1.30	0.08 × 10^5^	[54]
[Eu_2_(MFDA)_2_(HCOO)_2_(H_2_O)_6_ H_2_O	DMF	0.33	0.018	-	[55]
Tb^3+^@Cd-MOF	DMF	10	0.56	1.10 × 10^5^	[56]

^(a)^ LOD = limit of detection in two different units. ^(b)^ K_SV_ = Stern-Volmer constant.

## 3. Materials and Methods

Methyl-3-amino-4-bromobenzoate, 2-bromonaphthalene (98%), and (4-(methoxycarbonyl)phenyl)boronic acid (99.87%) were purchased from BLDpharm, and tetrakis(triphenylphosphine)palladium(0) (99%) and tri-tert-butyl phosphine (98%) from Sigma-Aldrich and used as received. Aluminum nitrate nonahydrate 98% was obtained from Roth. All solvents were bought from commercial suppliers with a minimum purity of 99.8% and used as received.

The ultrasonic device is a 2.8 L ultrasonic cleaner from Avantor (Avantor, Bruchsal, Germany). The oven used was a UNP 200–800 universal oven from Memmert (Memmert, Büchenbach, Germany). Fourier transform infrared spectroscopic measurements were performed using a Bruker Tensor 37 (Bruker AXS, Karlsruhe, Germany) with KBr pellets in the range between 4000 and 500 cm^−1^. For the N_2_ and CO_2_ sorption analysis, a Quantachrome Autosorb-IQ-MP (Quantachrome, Boynton Beach, FL, USA) was used. The samples were degassed for 24 h at 170 °C before the gas sorption measurements. The N_2_ sorption isotherms were collected at 77 K. The results were interpreted with the BET equation. The CO_2_ sorption temperatures were 293 and 273 K. The temperatures were held by virtue of a thermostated water bath (293 and 283 K) or with a Dewar filled with liquid N_2_ (77 K).

Thermogravimetric analysis was performed with a TG Tarsus 209 F3 (Netzsch, Selb, Germany). The samples were analyzed under synthetic air with a heating rate of 10 K/min from 25 to 1000 °C. Powder X-ray diffraction patterns were recorded using a Bruker D2 phaser benchtop diffractometer from Bruker (Bruker AXS, Karlsruhe, Germany) with Cu-Kα radiation, λ = 1.54182 Å at 300 W, 30 kV, and 10 mA. Nuclear magnetic resonance (^1^H-NMR) spectra were collected with a Bruker Avance III-600-I (Bruker, Karlsruhe, Germany). The chemical shifts are given in ppm and referenced to the residual proton signal of the solvent versus TMS (CDCl_3_: 7.26 ppm, DMSO-d6: 2.50 ppm). The excitation and photoluminescence spectra were measured with an FS5 spectrofluorometer from Edinburgh Instruments (Edinburgh, Scotland) with a 150 W CW ozone-free xenon arc lamp as a light source.

### 3.1. Synthesis of Dimethyl-2-Amino-[1,1′-biphenyl]-4,4′-Dicarboxylate [26]

In a three-neck 250 mL round-bottom flask, (4-(methoxycarbonyl)phenyl)boronic acid (40 mmol, 7.20 g) was mixed with methyl-3-amino-4-bromobenzoate (40 mmol, 9.20 g), tetrakis(triphenylphosphine)palladium (0) (1.8 mmol, 2.1 g), and potassium carbonate (180 mmol, 25 g) in 1,4-dioxane (160 mL) and water (40 mL). A clear solution was obtained in the flask when the mixture was refluxed at 100 °C for 24 h. The reaction was monitored via thin layer chromatography (TLC), and after completion, the reaction mixture was allowed to cool to room temperature, and the contents of the flask were filtered, and the resulting phase was extracted with 3 × 30 mL ethyl acetate. After separation, the organic phase was dried over magnesium sulfate for 15 min. The salt was then filtered, and the organic phase was concentrated using a rotary evaporator. The resulting red solid was dried overnight using a high vacuum oven at 60 °C. The crude product was purified by column chromatography using n-hexane: ethyl acetate (2:1 v:v) as eluent, resulting in the formation of white needle-like crystals. Yield = 6.7 g, 58%. ^1^H-NMR spectrum in Figure S1.

### 3.2. Synthesis of Dimethyl-2-(Naphthalen-2-ylamino)-[1,1′-biphenyl]-4,4′-Dicarboxylate

In a two-neck 100 mL round-bottom flask, 2-bromonaphthalene (3.8 mmol, 0.787 g, 1 eq) was dissolved in 40 mL of degassed dry toluene, and to this solution, 5 mol% each of palladium acetate (0.042 g) and tri-tert-butyl phosphine (0.05 mL) were added. This mixture was stirred under nitrogen for 10 min until the solution changed to a dark red color. After the color change, dimethyl-2-amino-[1,1’-biphenyl]-4,4’-dicarboxylate (4.5 mmol, 1.3 g, 1.2 eq) was further dissolved in 40 mL of toluene along with potassium carbonate (8 mmol, 1.1 g, 10 eq). The mixture was allowed to reflux at 111 °C for 24 h under thin-layer chromatography (TLC) monitoring. After cooling to room temperature, the contents were filtered, and the remaining solids were washed with ethyl acetate (3 × 50 mL). Afterwards, the ethyl acetate solution was washed with deionized water three times, with 50 mL each. After separation of the organic phase, consisting of toluene and ethyl acetate, it was dried over MgSO_4_ for 15 min, then concentrated using a rotary evaporator. The crude product was purified using flash column chromatography on silica gel 40–63 µm—Silicycle with a (v:v) 2:1 or 3:1 eluent of cyclohexane/ethyl acetate. The yield of the isolated yellow powder was 0.78 g, 50%. IR spectrum, Figure S10, ^1^H-NMR, Figure S2.

The up-scaled reaction of this compound was performed as follows: In a two-neck 1 L round-bottom flask, 2-bromonaphthalene (24.7 mmol, 5.116 g, 1 eq) was dissolved in 260 mL of degassed dry toluene. To this solution, 5 mol% each of palladium acetate (0.273 g) and tri-tert-butylphosphine (0.325 mL) were added. The mixture was stirred under nitrogen for 10 min until the solution turned dark red. After the color change, dimethyl-2-amino-[1,1′-biphenyl]-4,4′-dicarboxylate (29.25 mmol, 8.45 g, 1.2 eq) was dissolved in another 260 mL of toluene, along with potassium carbonate (52 mmol, 7.15 g, 10 eq). The reaction mixture was then refluxed at 111 °C for 24 h, under TLC monitoring. After cooling to room temperature, the contents were filtered, and the remaining solids were washed with ethyl acetate (3 × 100 mL). The combined organic phase was then washed with deionized water (3 × 100 mL). The organic layer (toluene + ethyl acetate) was dried over MgSO_4_ for 15 min, then concentrated using a rotary evaporator. The crude product was purified by flash column chromatography on silica gel (40–63 µm, Silicycle), using a 2:1 or 3:1 v:v mixture of cyclohexane/ethyl acetate as eluent. The isolated product was obtained as a yellow powder. The yield was 5.3 g, 52%. The IR and ^1^H-NMR spectra were identical to those in Figures S10 and S2.

### 3.3. Synthesis of 2-(Naphthalen-2-ylamino)-[1,1′-biphenyl]-4,4′-Dicarboxylic Acid

In a 100 mL round-bottom flask, dimethyl-2-(naphthalen-2-ylamino)-[1,1′-biphenyl]-4,4’-dicarboxylate (3.0 g, 7.2 mmol) was dissolved in 40 mL of THF. To this solution, 40 mL of NaOH (1 mol L^–1^) solution was added and refluxed for 24 h at 100 °C. After cooling down to room temperature, THF was removed using a rotary evaporator. To the aqueous phase, 40 mL of a 0.1 mol L^–1^ HCl solution was added. The yellow solid was filtered and dried under vacuum at 80 °C. Yield = 2.49g, 90%. IR spectrum Figure S10, ^1^H-NMR Figure S3.

### 3.4. Synthesis of the Al-BP-Naph

2-(Naphthalen-2-ylamino)-[1,1′-biphenyl]-4,4′-dicarboxylic acid (0.2 mmol, 0.076 g) and aluminum nitrate nonahydrate (0.2 mmol, 0.075 g) were dissolved in 6 mL of DMF with the assistance of the ultrasound device. The reaction mixture was heated to 110 °C over 1 h, held at this temperature for 24 h, and allowed to cool to room temperature within 1 h. The precipitated powder was separated by centrifugation and subsequently washed with 40 mL of DMF via a treatment at 80 °C for 24 h in a flask with occasional mild stirring to avoid mechanical deterioration of the product. The product was separated by centrifugation and dried at 70 °C for 24 h using a vacuum oven (1 × 10^−3^ bar). The yield after the washing and drying was 119 mg, 80%.

### 3.5. Quenching Experiments

To evaluate the quenching selectivity and sensitivity of Al-BP-Naph, suspensions were prepared in DMF at concentrations of 1 g L^−1^ for selectivity studies and 0.1 g L^−1^ for sensitivity measurements. In the selectivity experiments, various metal nitrate solutions (c = 0.1 mol L^−1^) were employed from which 100 µL were added to the 3.0 mL of the Al-BP-Naph DMF suspension to give a metal ion concentration of 0.003 mol L^−1^. In the sensitivity experiments, the concentration of the added iron(III) nitrate solution was 0.001 mol L^–1^, from which 10 µL aliquots were added to the Al-BP-Naph suspension

For each measurement, 3.0 mL of the Al-BP-Naph suspension was transferred into a thoroughly cleaned quartz cuvette. The initial emission intensity of the suspension was recorded as a reference (blank) prior to the incremental addition of the analytes in steps of 10, 25 or more µL in the sensitivity experiments. All experiments were conducted under identical conditions to ensure maximum reproducibility and comparability of results.

## 4. Conclusions

In this work, we present a luminescence sensor based on a functionalized MOF with a MIL-53 or DUT-5 topology bearing a naphthylamino chromophore group on the linker. Al-BP-Naph exhibits a surface area of 465 m^2^ g^−1^ and a total pore volume of 0.546 cm^3^ g^–1^, with a zero-coverage isosteric heat of CO_2_ adsorption of 26 kJ mol^−1^. Contrary to the solid MOF, the suspended MOF in DMF shows a slight red shift due to the solvatochromic effect. The MOF displays exceptional luminescence sensitivity for the detection of Fe^3+^ ions with a rapid turn-off response and a remarkably high Stern-Volmer constant of 9000 L mol^−1^ when we excited the MOF suspension at 415 nm and monitored the luminescence at 520 nm. Our findings indicate that Fe^3+^ in solution competes for the excitation energy required for the luminescence of Al-BP-Naph, effectively quenching the MOF’s emission through a competitive energy absorption mechanism. This quenching effect is highly selective, as no significant response was observed for other metal ions. The fast response within 10 s to iron excludes the possibility of any complex formation causing static quenching. Similar ionic radii of other tested metal ions compared to Fe^3+^ exclude dynamic quenching.

Furthermore, the sensitivity of the MOF toward Fe^3+^ is seen with a concentration lower than 5.6 × 10^–6^ mol L^–1^. The calculated limit of detection (LOD) of 5.6 × 10^–6^ mol L^–1^ (rounded to 6 × 10^–6^ mol L^–1^) highlights its potential as a highly efficient iron nitrate sensor. These combined results establish Al-BP-Naph as a promising candidate for practical applications in luminescent Fe^3+^ sensing.

## Data Availability

The original contributions presented in this study are included in the article/Supplementary Material. Further inquiries can be directed to the corresponding author(s).

## Supplementary Materials

The following supporting information can be downloaded at https://www.mdpi.com/article/10.3390/molecules30204146/s1: Section S1: Reaction schemes for ligand synthesis (Scheme S1–S3) and nuclear magnetic resonance spectra; Section S2: Structure determination and PXRD pattern; Section S3: Infrared spectroscopy; Section S4: Thermogravimetric analysis; Section S5: Digestion NMR analysis; Section S6: N_2_ adsorption; Section S7: CO_2_ adsorption and isosteric heat (enthalpy) of adsorption; Section S8: Photoluminescent properties; Section S9: References. Reference [57] is cited in the Supplementary Materials.
