# Correction: Othman et al. Synthesis of a Luminescent Aluminum-Based MOF for Selective Iron(III) Ion Sensing. *Molecules* 2025, *30*, 4146

**DOI:** 10.3390/molecules30234540

**Published:** 2025-11-25

**Authors:** Hanibal Othman, István Boldog, Christoph Janiak

**Affiliations:** Institut für Anorganische Chemie und Strukturchemie, Heinrich-Heine-Universität Düsseldorf, D-40204 Dusseldorf, Germany; hanibal.othman@hhu.de (H.O.); Istvan.Boldog@hhu.de (I.B.)

## Error in Figure

In the original publication [1], there was a mistake in Figure 5b. The published Figure 5b was the same as Figure 5a. During typesetting, Figure 5b was omitted and Figure 5a duplicated. This error was not noted during the proofreading. The published figure caption in [1] is repeated below and referred to the corrected Figure 5b. The corrected Figure 5b appears below. The authors state that the scientific conclusions are unaffected. This correction was approved by the Academic Editor. The original publication has also been updated.

## Figures and Tables

**Figure 5:**
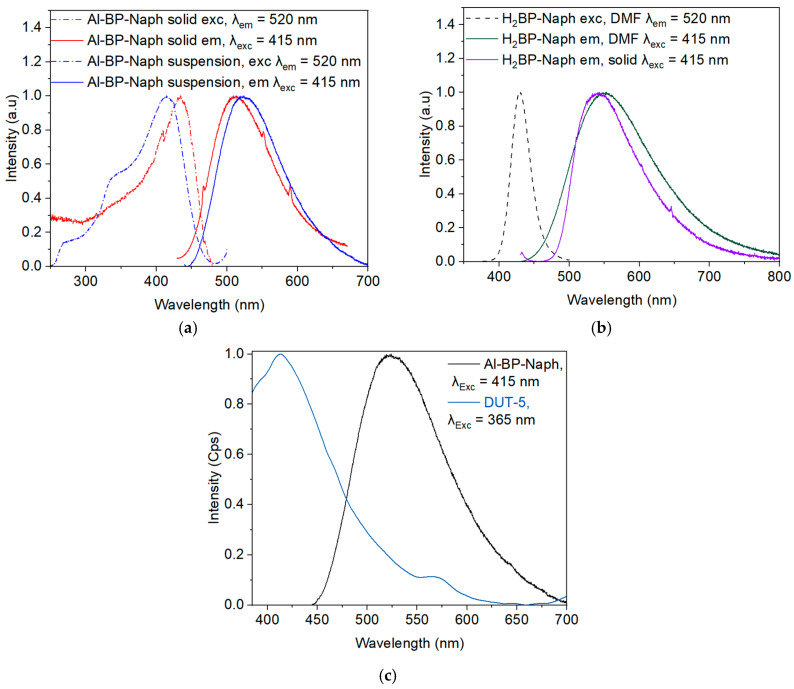
(**a**) Excitation (dashed lines) and steady-state photoluminescence spectra (solid lines) of Al-BP-Naph in solid form (red) and suspended (blue curves) in DMF. (**b**) H_2_BP-Naph linker excitation and emission spectrum in DMF and the solid state. (**c**) Emission spectra of Al-BP-Naph and DUT-5, where Al-BP-Naph was excited at λ_exc_ = 415 nm and DUT-5 at λ_exc_ = 365 nm.
