# Supplementary material for: Synthesis of a Luminescent Aluminum-Based MOF for Selective Iron(III) Ion Sensing
Source: Molecules. 2025 Oct 21;30(20):4146. doi: 10.3390/molecules30204146 (PMC12566859; doi:10.3390/molecules30204146)
Supplement: Supplementary file 1 [file molecules-30-04146-s001.zip › molecules-3897349-supplementary.pdf]

# Synthesis of a Luminescent Aluminum-Based MOF for Selective Iron(III) Ion Sensing

**Hanibal Othman, István Boldog and Christoph Janiak ***

Institut für Anorganische Chemie und Strukturchemie, Heinrich-Heine-Universität Düsseldorf, D-40204 Dusseldorf, Germany
* Correspondence: janiak@uni-duesseldorf.de

**Content**

**Section S1. Reaction schemes for ligand synthesis (Scheme 1-3) and nuclear magnetic resonance spectra S2**

**Section S2. Structure determination and PXRD pattern S5**

**Section S3. Infrared spectroscopy S10**

**Section S4. Thermogravimetric analysis S11**

**Section S5. Digestion NMR analysis S12**

**Section S6. $N_2$ adsorption S13**

**Section S7. $CO_2$ adsorption and isosteric heat (enthalpy) of adsorption S14**

**Section S8. Photoluminescent properties S16**

**Section S9. References S18**

**Section S1. Reaction schemes for ligand synthesis (Scheme 1-3) and nuclear magnetic resonance spectra**

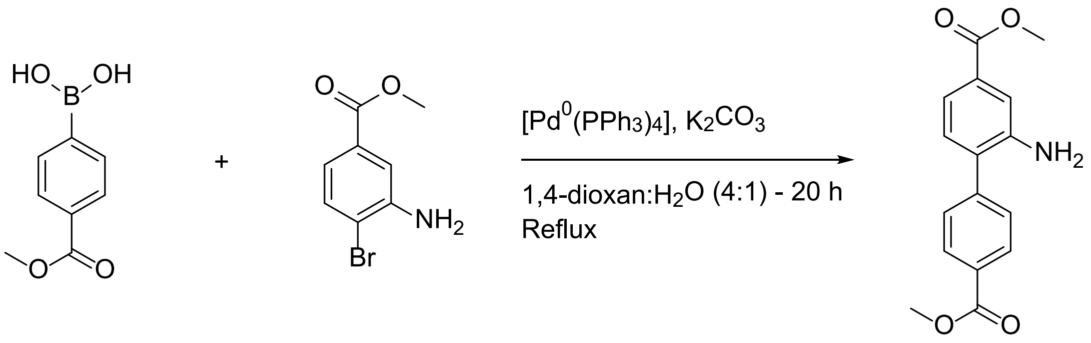


**Scheme S1**. Synthesis of dimethyl-2-amino-[1,1'-biphenyl]-4,4'-dicarboxylate [1].

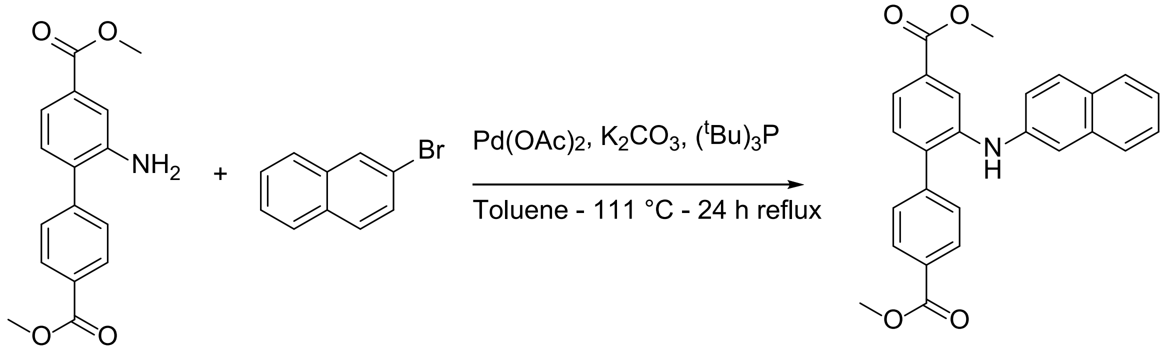


**Scheme S2**. Synthesis of dimethyl-2-(naphthalen-2-ylamino)-[1,1'-biphenyl]-4,4'-dicarboxylate.

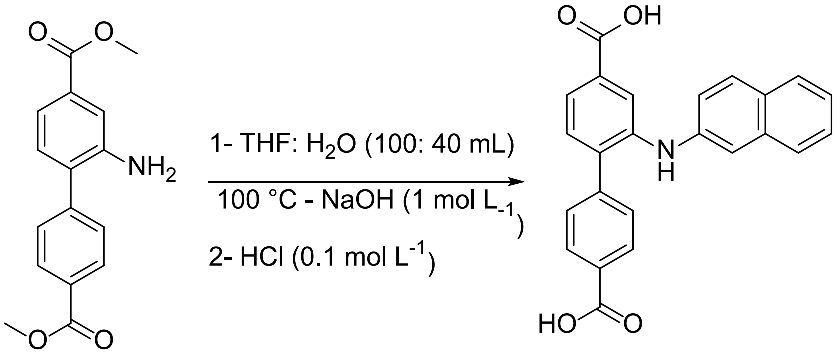


**Scheme S3**. Synthesis of 2-(naphthalen-2-ylamino)-[1,1'-biphenyl]-4,4'-dicarboxylic acid ($H_2$BP-Naph).

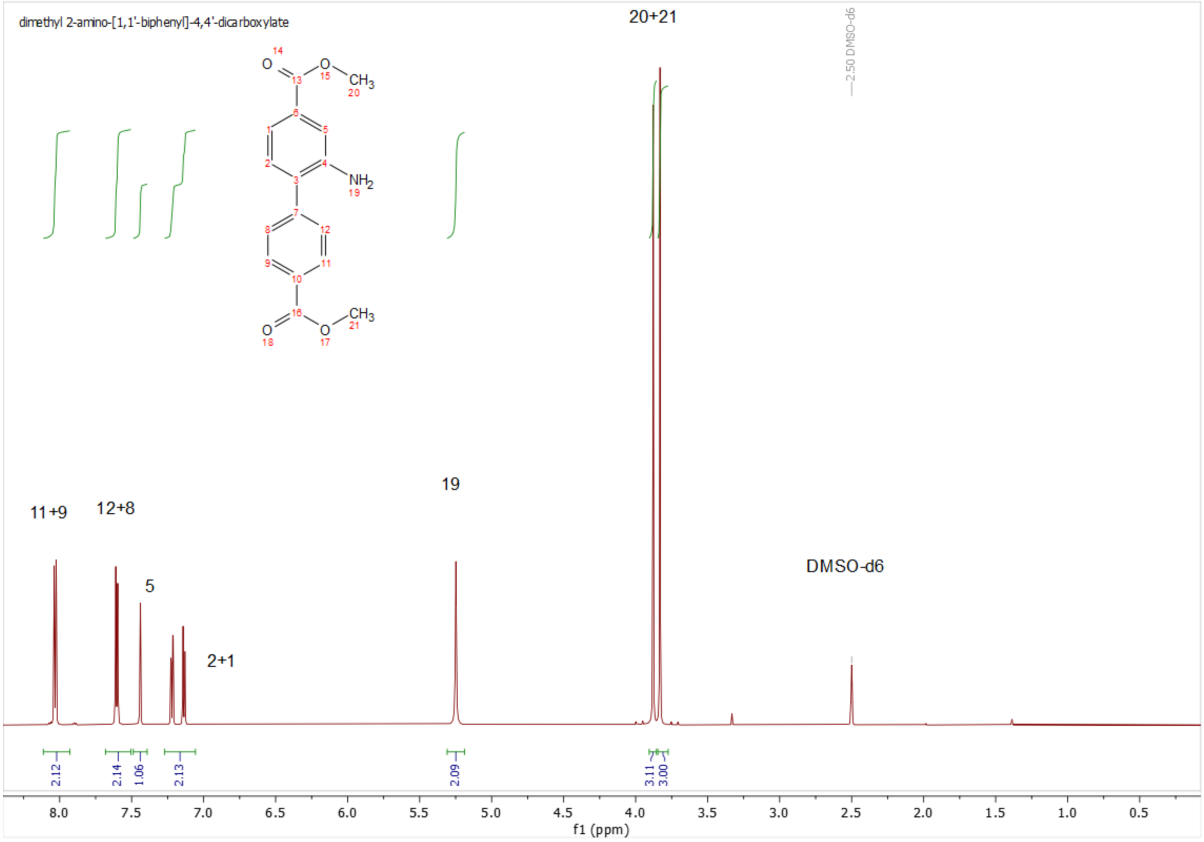


**Figure S1**. $^{1}H$ NMR spectrum (600 MHz, DMSO-d6) of dimethyl-2-amino-[1,1'-biphenyl]-4,4'-dicarboxylate.

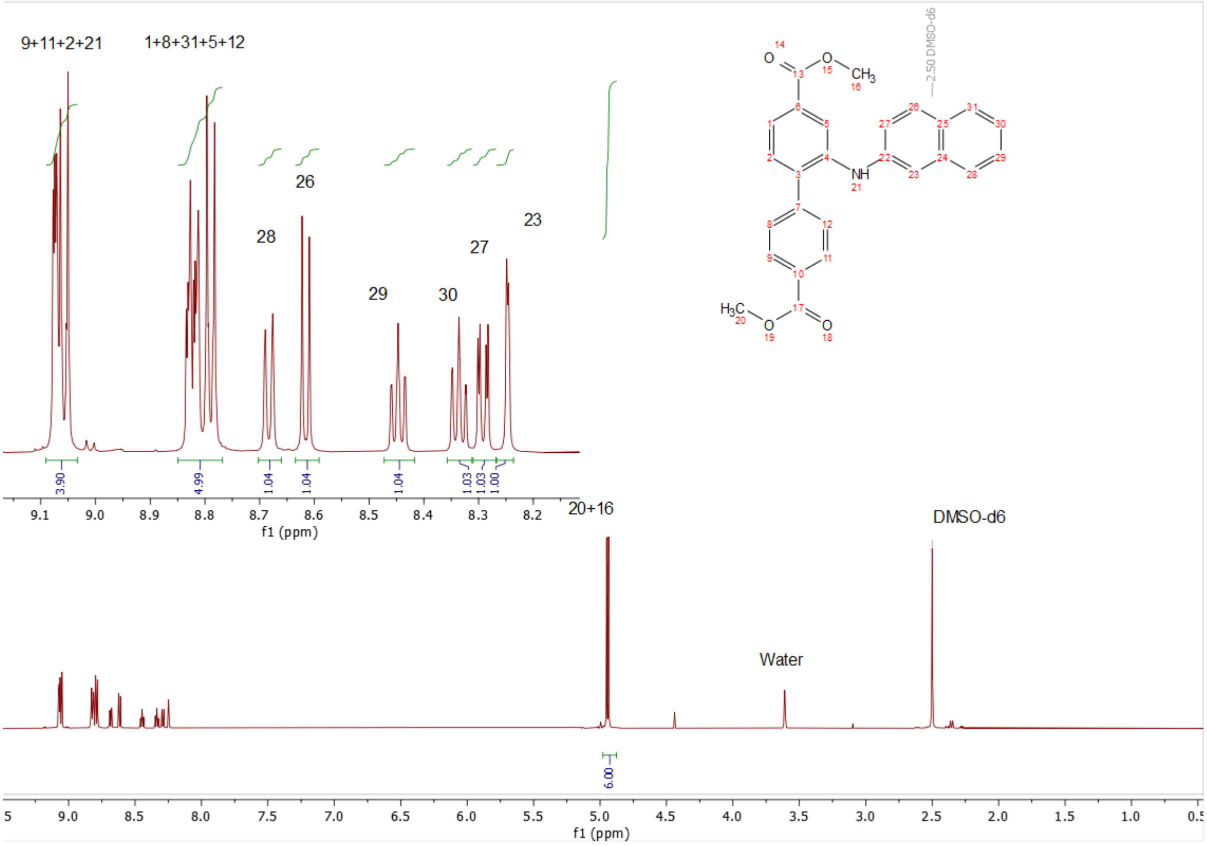


**Figure S2**. $^{1}H$ NMR spectrum (600 MHz, DMSO-d6) of dimethyl- 2-(naphthalen-2-ylamino)-[1,1'-biphenyl]-4,4'-dicarboxylate ($Me_2BP$-Naph).

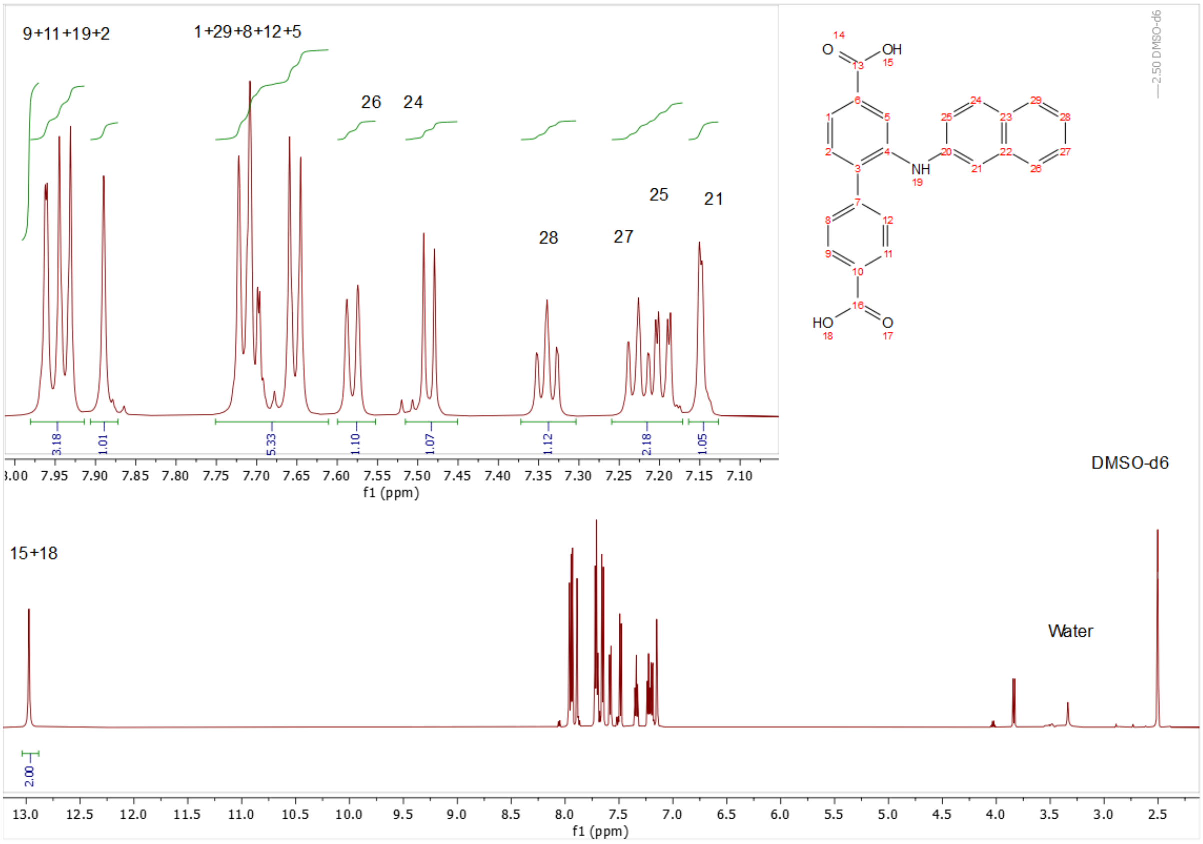


**Figure S3**. $^{1}$H NMR (600 MHz, solvent DMSO-d6) NMR spectrum of 2-(naphthalen-2-ylamino)-[1,1'-biphenyl]-4,4'-dicarboxylic acid $H_2$BP-Naph.

**Section S2. Structure determination and PXRD pattern**

Le Bail fit*:*

The Powder X-ray diffraction (PXRD) data was Le Bail-fitted using the space group and the initial cell parameters for the published structure of [VO(BPDC)], where $H_2BPDC$ = 4,4′-biphenyldicarboxylic acid, also known as COMOC-2-lp (lp stands for large pore) [2]. The Le Bail profile fitting yields excellent results (**Figure S1**, **Table S1**), however they are rather an indication of just moderate crystallinity (still excellent for such a case) and, hence, strong peak broadening, which allows precise fit, but provides little structural information.

Notably, the cell parameters of Al-BP-Naph significantly differ from those of COMOC-2-*lp*. While the cell volumes are nearly the same (~3% difference), the structure of Al-BP-Naph features as slight deflection from the nearly fully opened structure of COMOC-2-*lp*, which has nearly square shaped the lozenge pores running along the *b*-axis (*a/c* = 1.18 vs 1.04 respectively), while the fully closed structure is characterized by maximally elongated „narrow" lozenge pores.

It is presumed, that the naphthylaminyl substituent of the ligand in Al-BP-Naph is heavily disordered. As the ligand is essentially non-planar due to steric reasons, the possible and highly probable disorder on four positions (formally corresponding to 2,2',6,6' locants) is most probably complemented by the disorder due to the flexibility of the ligand, constituted of non-conjugated aromatic moieties. The moieties have a limited rotational degree of freedom, which could give several conformations with local energy minima, depending on the realized sets of intermolecular interactions with the neighboring „walls" of the framework and/or another naphthylaminyl subsituents. The latter interactions should be held responsible for the observed deflection of the cell parameters from the case of most opened structure. An attempt of a Rietveld refinement confirms the „smeared" electron densities at the ligands's localization, which precluded the possibility of a proper structural refinement.

The single naphthylaminyl substituent per ligand does not occupy the whole space of the pore leaving pores large enough to allow the adsorption of $N_2$ molecules (see Figure S5 for an arbitrarily chosen possible substituent localization).

The PXRD data for Al-BP-Naph was collected by a Rigaku Miniflex PXRD diffractometer using Cu-$K\alpha_{1,2}$ radiation in a Bragg-Brentano geometry on a flat sample employing a low-background silicon sample-holder with an indent. The sample was thoroughly homogenized, but no special grounding or sieving were used. The measurement was performed in air during 8.5h with 0.01° 2θ angular steps.

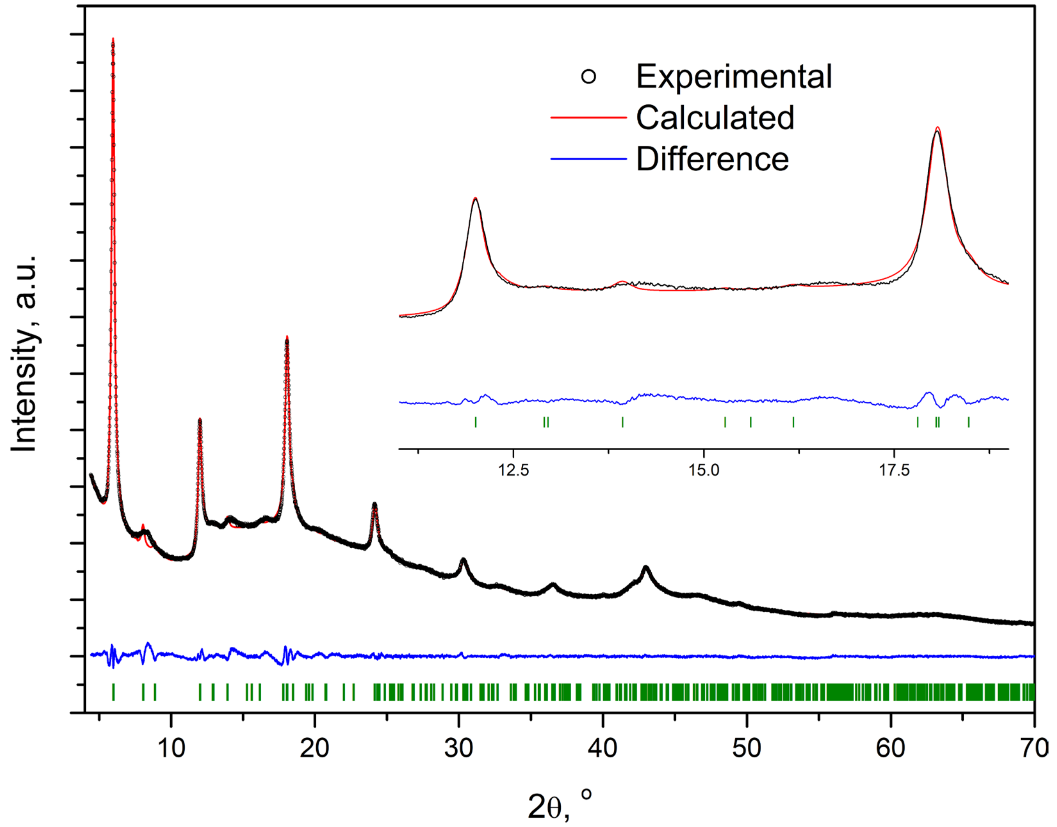


**Figure S4**. Le Bail fit of the PXRD data for Al-BP-Naph.

**Table S1**. Le Bail fitting results for Al-BP-Naph and its comparison with the reported crystal data and refinement details for the prototype structure COMOC-2-lp.

| | **COMOC-2-lp** | **Al-BP-Naph** |
|---|---|---|
| **Diffractometer** | STOE STADI P | Rigaku Miniflex 600 (2019) |
| **Temperature / K** | 293(2) | 295(2) |
| **Wavelength / Å** [a] | 1.5418 | 1.54051, 1.54433 [a] |
| **Crystal system** | Orthorombic | Orthorombic |
| **Space group** | *I*mma | *I*mma (disorder is implied) |
| ***a*** **/Å** | 21.443(3) | 21.901(3) |
| ***b*** **/Å** | 6.957(4) | 7.2686(13) |
| ***c*** **/Å** | 20.570(2) | 18.5134(4) |
| ***V*** **/Å³** | 3069(5) | 3168.8(9) |
| $R_p$, $wR_p$ | 0.035, 0.0507 | 0.0137, 0.0191 |
| ***R, wR [I>3σ], Rietveld*** | 0.0875, 0.0640 | - |
| **Goodness-of-fit,** $\chi^2$ | n.d. | 0.91 |

[a] Cu K$\alpha_1$,$\alpha_2$; I(K$\alpha_2$)/I(K$\alpha_1$) = 0.5

The Le Bail fit was performed using the Jana 2006 software [2] (note that the Le Bail fit, unlike the Rietveld fit, does not involve the information about the actual structure, but uses only the cell dimensions and space group. The Le Bail profile fit demonstrate the adequacy of the chosen space group and cell parameters combination for the given data). Manual background was used, while the three cell parameters for the assumed orthorhombic cell (*I*mma) symmetry in analogy with the published structure of COMOC-2-lp [3], two Lorenzian peak shape function parameters, the Simpson asymmetry correction parameter, and the sample shift were refined. The summary together with the comparable data for the COMOC-2-lp is given in **Table S1**.

It is worth noting that the disorder or the naphthylaminyl substituent precludes the precise determination of the space group. The *I*mma space group observed in COMOC-2-lp was retained, however even in the latter case, with structure solution and refinement based on powder data, the actual space group might be incorrect. Nevertheless, the primary objective of the PXRD data analysis was to confirm the structure of the framework, rather than attempt a Rietveld refinement of a seemingly strongly disordered structure using a data collected on a sample with just a moderate crystallinity (the strong disorder and compromised crystallinity are to a significant degree correlated). The difference Fourier maps for an attempted Rietveld refinement using COMOC-2-lp input with pre-refined cell parameters confirmed the placement of the metal atoms, however. the electronic density associated with the ligand was too smeared, making the pursue of the refinement unreasonable.

To demonstrate a possible localization of the naphthylaminyl pendant group a structural model was built manually, and the result, shown on **Figure S5**-Figure S7 should rather be viewed as a semi-artistic representation, even though the geometry of the cell and the molecular sizes are faithfully held to demonstrate the residual porosity. A lowered *I*m11 symmetry was chosen as one of the few possibilities; while it is not the highest apparent symmetry for a periodic structure with localized ligand's substituents, but it allows an easy demonstration of the residual porosity.

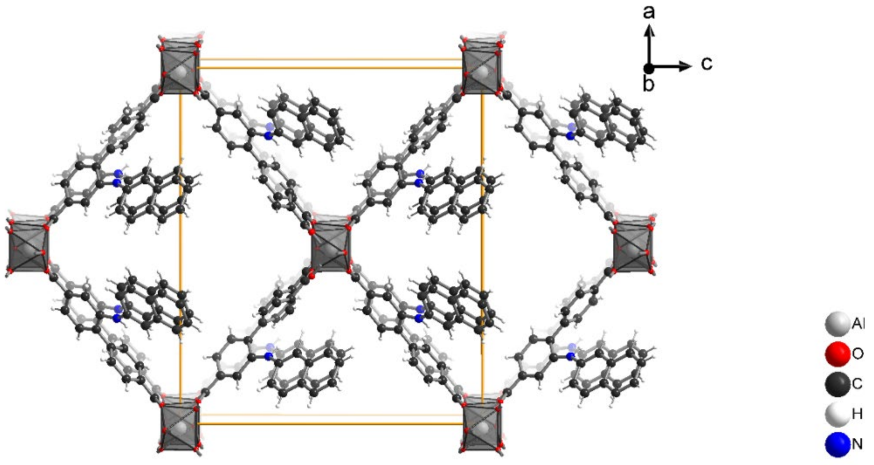


**Figure S5**. The approximate possible localization of the disordered naphthylamino pendant group in Al-BP-Naph (Im11 space group)

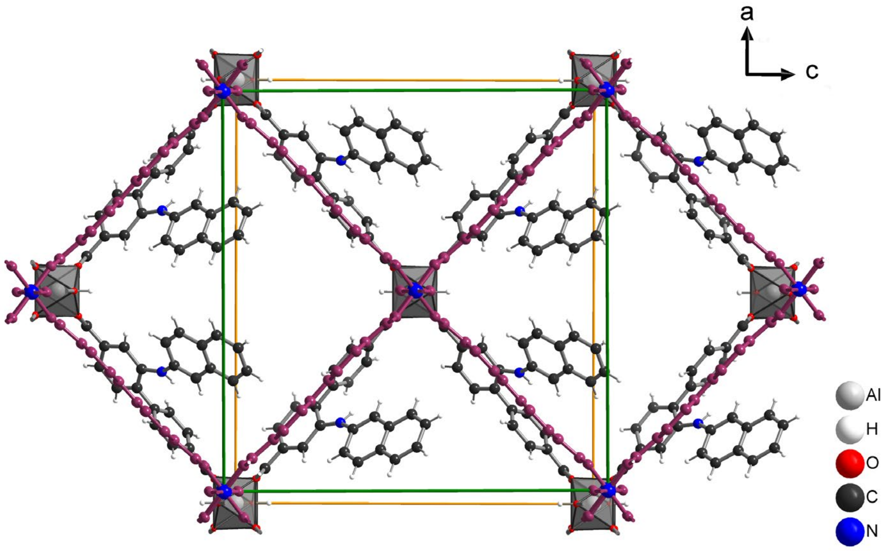


**Figure S6**. Overlap of the COMOC-2-lp cell and the model of Al-BP-Naph along the b axes with one of the possible localizations of the naphthylaminyl pendant group (Im11 space group).

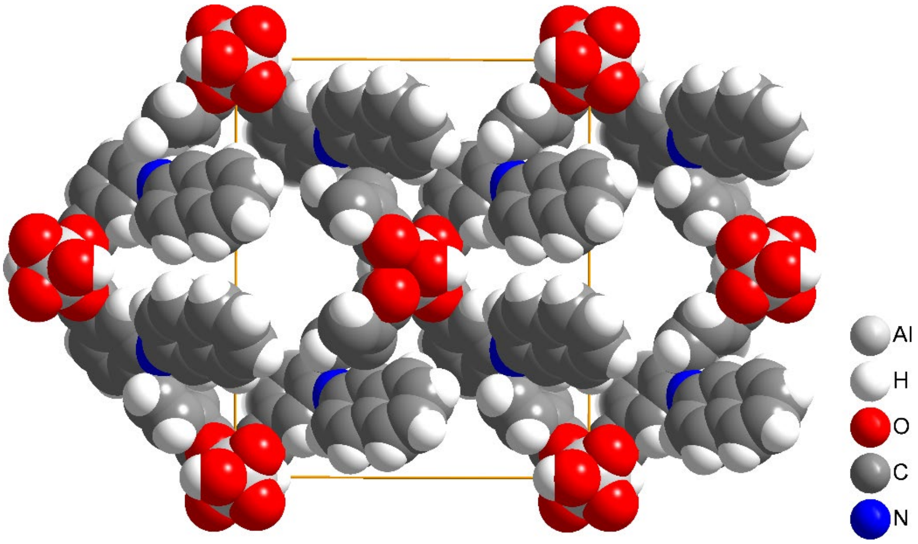


**Figure S7**. Space filling model for a structure representing a possible localization of the disordered naphthylamino pendant group in Al-BP-Naph (Im11 space group). Note the 4x4 Å$^2$ pore entrances, which appear barely accessible, but allow the adsorption of the $N_2$.

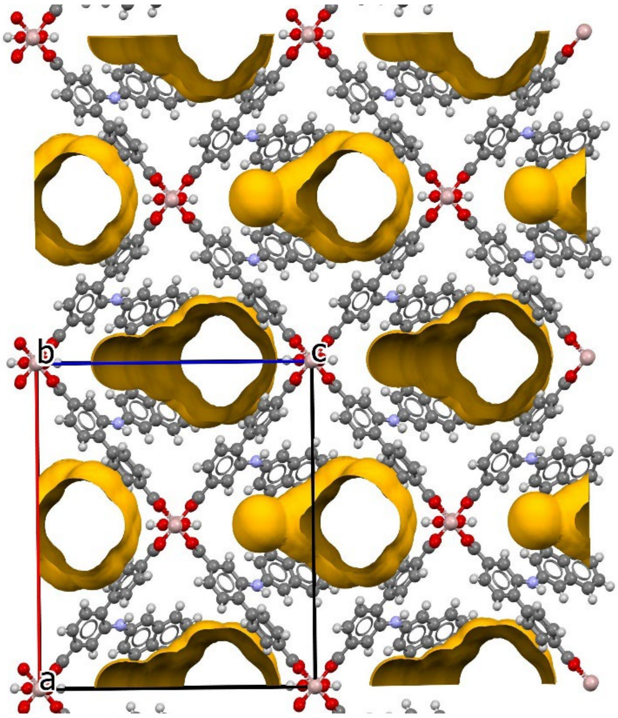


**Figure S8**. The model of the structure of S3, based on the comparison of PXRD with analogous COMOC-2-lp and DUT-5 structures with resolved naphthylamino-group disorder with one fixed position among the range of possible (the structure is given in lower-symmetry I).

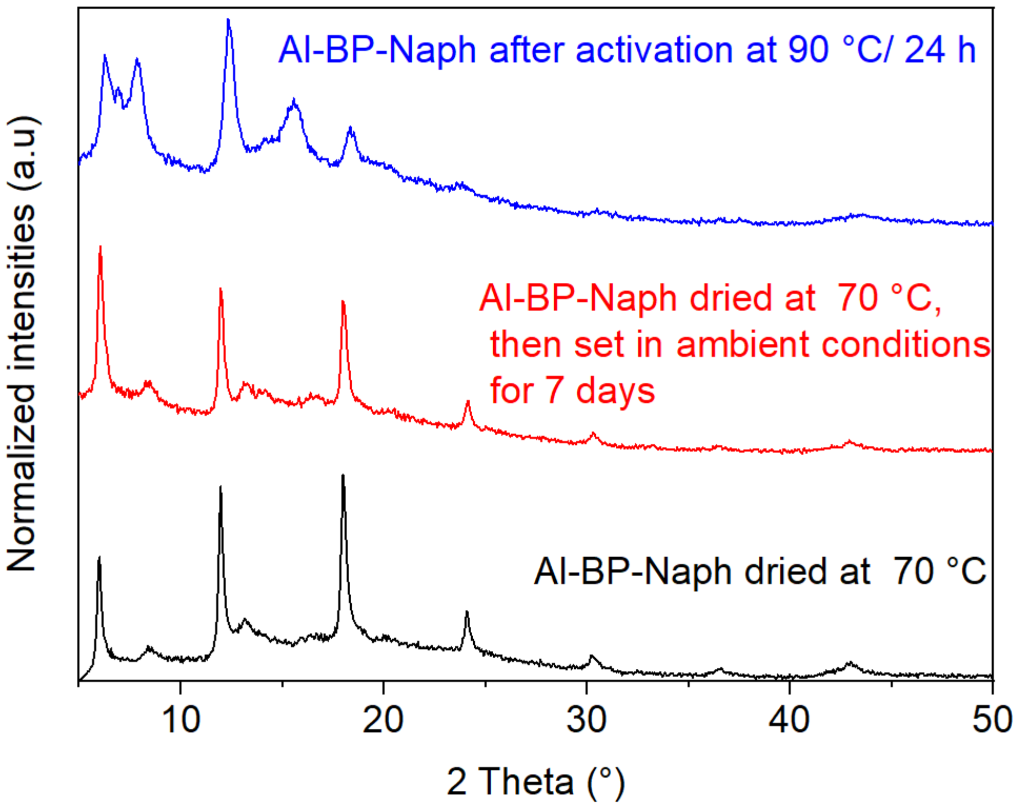


**Figure S9**. PXRD of dried Al-BP-Naph at 70 °C (black), dried Al-BP-Naph after 7 days in ambient conditions (red) and activated Al-BP-Naph (Blue).

**Section S3. Infrared spectroscopy**

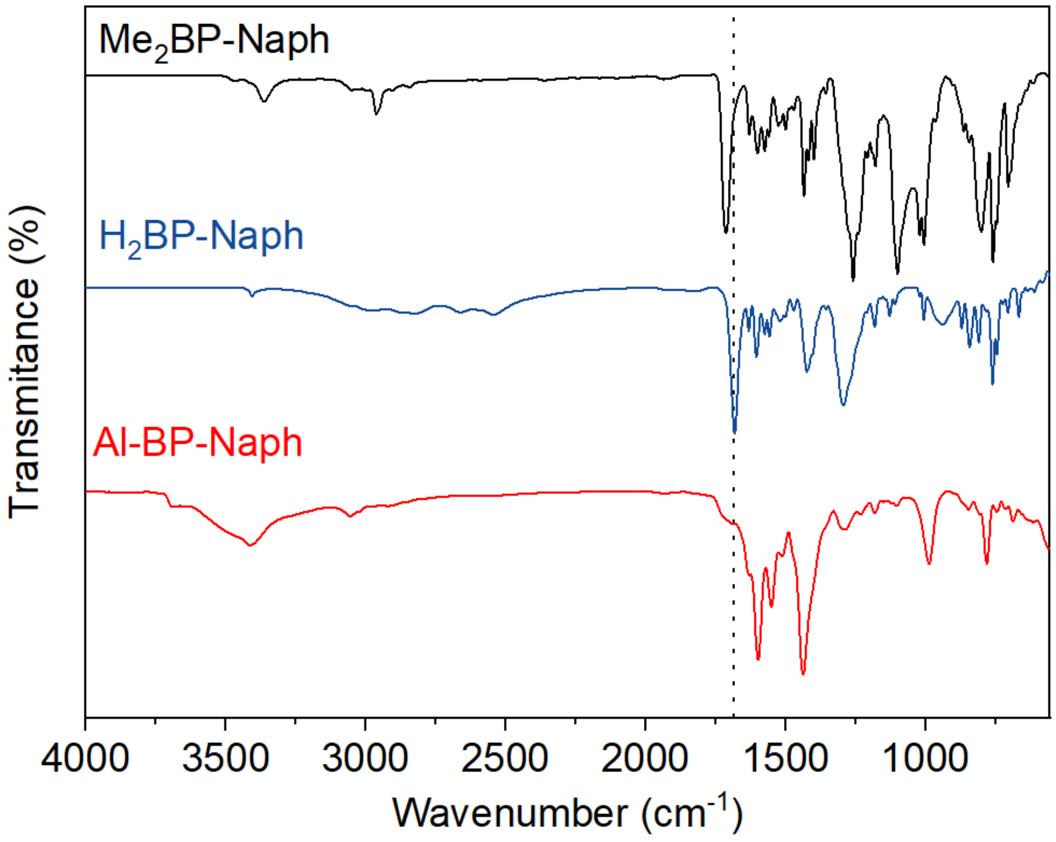


**Figure S10**. IR of Al-BP-Naph (red), free acid $H_2BP$-Nach linker (navy blue) and methylated linker $Me_2BP$-Naph (black) as KBr pellets.

**Section S4. Thermogravimetric analysis**

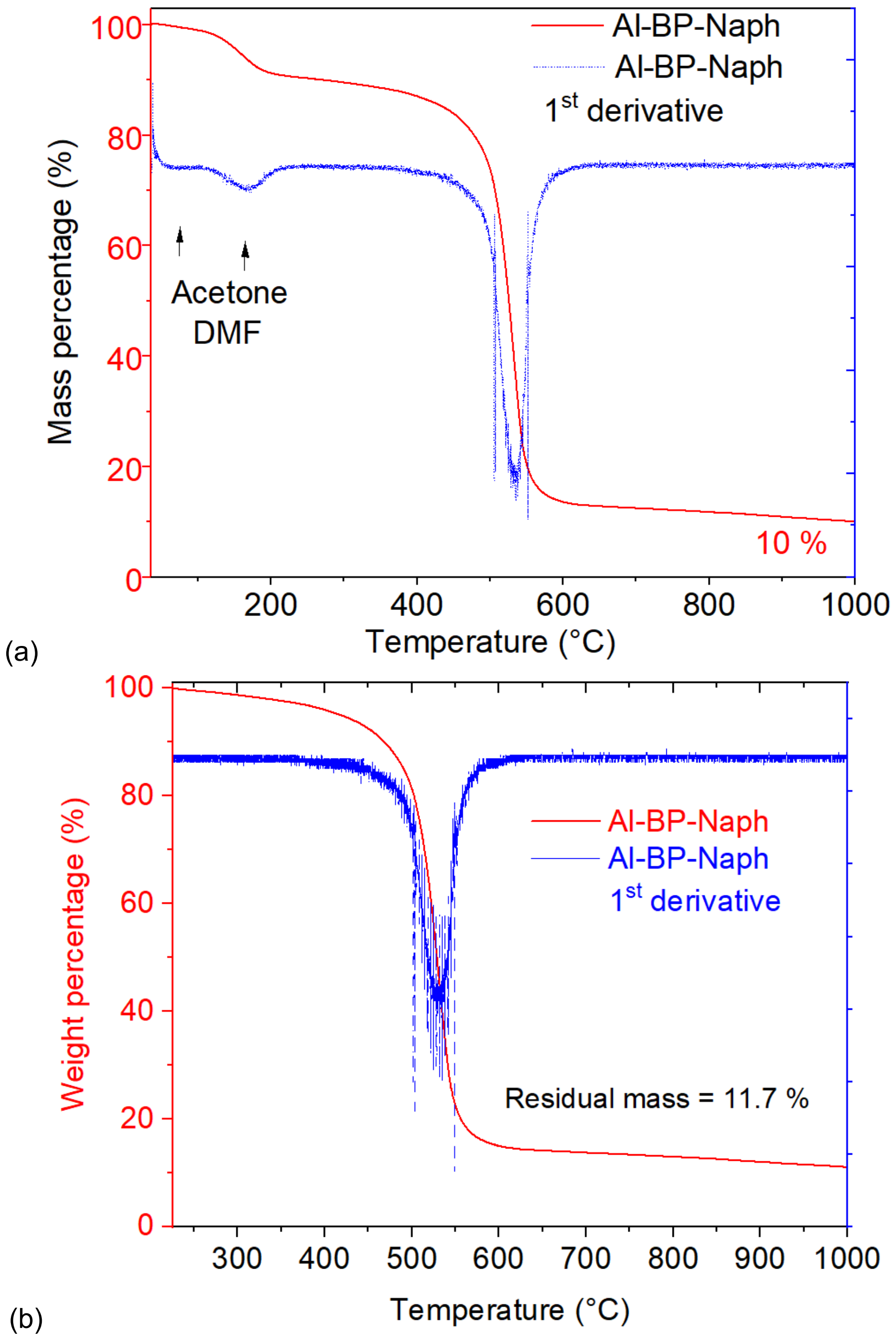


**Figure S11**. (a) TGA of Al-BP-Naph under synthetic air with a heating rate of 10 K $min^{-1}$. (b) Rescaled TGA plot after the solvent loss at 200 °C with the mass at 225 K set to 100%.
The thermogravimetric analysis was done with the NETZSCH TG 209 F3 Tarsus under synthetic air.

The theoretical mass loss of the BP-Naph linker from Al-BP-Naph is calculated to 89.7 wt% and the residual mass of $Al_2O_3$ to 11.98 wt%.

Al-BP-Naph has the molecular formula of [Al(OH)(BP-Naph)], $AlC_{24}H_{16}NO_5$.
$M_{Al\text{-}BP\text{-}Naph}$ = 425.38 g $mol^{-1}$
$M_{Al}$ = 26.98 g $mol^{-1}$
$M_{BP\text{-}Naph}$ = 381.39 g $mol^{-1}$
1 mol of Al-BP-Naph gives 0.5 mol $Al_2O_3$ (1/2 $M_{Al2O3}$ = 50.98 g $mol^{-1}$)
50.98/425.38 × 100% = 11.98 % theoretical percentage of $Al_2O_3$ in Al-BP-Naph

## Section S5. Digestion NMR analysis

TGA data already supports the composition of the ligand within Al-BP-Naph.
Yet, to confirm the integrity of the organic BP-Naph ligand within the MOF structure we have carried out a digestion NMR analysis.
In an NMR tube 10 mg of Al-BP-Naph was placed in 100 µL of NaOD/$D_2O$ (1 mol/L) and shaken until a clear solution was obtained. Then 0.7 mL of DMSO-$d_6$ was added and the digestion NMR spectrum in Figure S12 showed all signals for the BP-Naph ligand (cf. Figure S3), albeit slightly shifted because of the deprotonation in the alkaline solution.

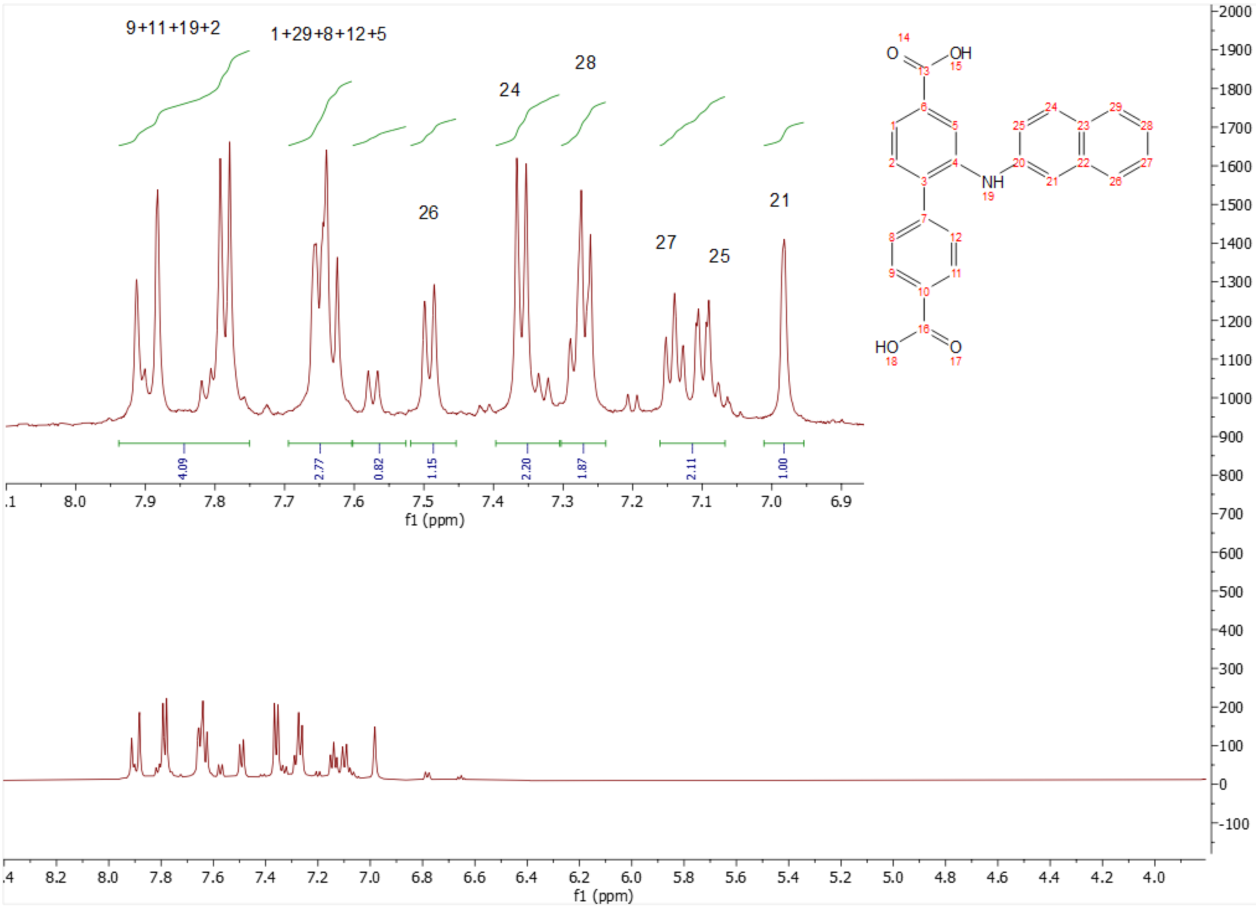


**Figure S12**. ("Digestion") $^1$H NMR spectrum (600 MHz, solvent NaOD/$D_2O$/DMSO-$d_6$) of the solid Al-BP-Naph dissolved in NaOD/$D_2O$/DMSO-$d_6$ to verify the intact linker 2-(naphthalen-2-ylamino)-[1,1'-biphenyl]-4,4'-dicarboxylate. BP-Naph$^{2-}$ (deprotonated in the alkaline medium).

## Section S6. $N_2$ adsorption

The specific Brauner-Emmett-Teller surface area $S_{BET}$ was calculated using the following equation (1)

$$S_{BET} = v_m \cdot N_A \cdot A_m / V_m \quad (1)$$

Where: $S_{BET}$ = BET surface area $m^2\ g^{-1}$
$v_m$ = Monolayer volume $cm^3\ g^{-1}$
$N_A$ = Avogadro's number ($6.022 \cdot 10^{23}$ molecules $mol^{-1}$)
$A_m$ = Cross-sectional area of the adsorbate molecule (0.162 nm² for $N_2$)
$V_m$ = Molar volume of ideal gas a STP = 22.414 L $mol^{-1}$

$$\frac{1}{V\left(\frac{P_0}{P}-1\right)} = \frac{C-1}{(V_m C)} \cdot \frac{P}{P_0} + \frac{1}{V_m C} \quad (2)$$

where:
V = Volume of gas adsorbed at pressure P ($cm^3\ g^{-1}$)
$V_m$ = Monolayer capacity (volume of gas required to form a monolayer, cm³/g)
P = Partial pressure of the adsorbate gas
$P_0$ = Saturation pressure of the adsorbate gas
C = BET constant, related to the energy of adsorption ($e^{\frac{E_1 - E_L}{RT}}$)
where:
$E_1$ = heat of adsorption for the first layer
$E_L$ = heat of vaporization

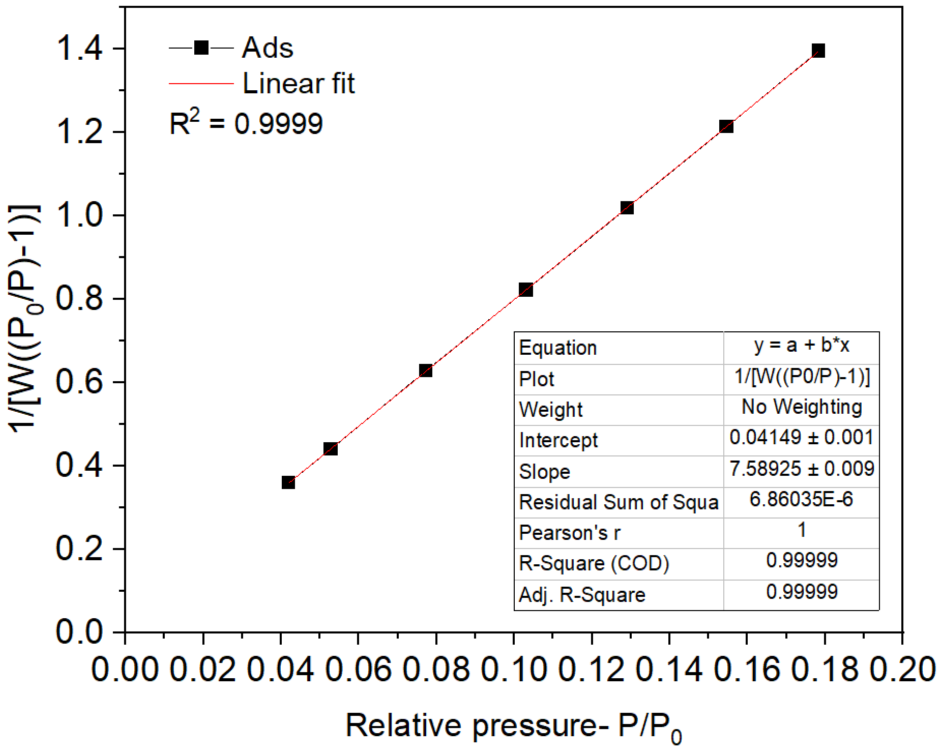


**Figure S13**. Derivation of the BET surface area from the 77 K nitrogen adsorption isotherms for Al-BP-Naph.

**Section S7. $CO_2$ adsorption and isosteric heat (enthalpy) of adsorption**

The isotherms were collected using a QUANTACHROME Autosorb-iQ-MP. The isosteric heat of adsorption was calculated using two different sets of isotherm data points at the different temperatures of 293 K and 273 K using the following Freundlich-Langmuir method with equation (2)

$$n = a \cdot b \cdot p^c / 1 + b \cdot p^c \qquad (2)$$

Where n: the adsorbed amount (mmol $g^{-1}$).

a: the maximal loading (mmol $g^{-1}$)

b: the affinity constant

c: the heterogeneity exponent

The pressure at the given adsorbed amount can be calculated when rearranging the Freundlich-Langmuir equation to the following form, equation (3).

$$P(n) = c \cdot \sqrt{(n / a \cdot b - n \cdot b)} \qquad (3)$$

The parameters a, b and c were taken from the fitting equation.

The isosteric enthalpy of adsorption was then calculated via the Clausius-Clapeyron equation (4):

$$\Delta H_{(ads)} \cdot n = - R \cdot \ln(p_2/p_1) \cdot (T_1 \cdot T_2)/(T_2 - T_1) \qquad (4)$$

Where $\Delta H_{(ads)}$: the isosteric enthalpy of adsorption kJ $mol^{-1}$

$T_1$: absolute Temperature in K (here 273 K)

$T_2$: absolute Temperature in K (here 293 K)

R: the universal gas constant with the value 8.3145 J $K^{-1}$ $mol^{-1}$.

According to Nuhnen et al. [4], when interpolating the loading n, the heat of adsorption $Q_{st}$ is obtained as a function of a loading (equation (5)-(7)).

$$\Delta H_{(ads)} = - R \cdot m' \qquad (5)$$

$$m' = \ln(p_2/p_1) \cdot (T_1 \cdot T_2)/(T_2 - T_1) \qquad (6)$$

$$\Delta H_{(ads)} = - Q_{st} \qquad (7)$$

**Table S2**. Fitting constants of the Freundlich-Langmuir fit.

| Measurement | A | B | C | $R^2$ |
|---|---|---|---|---|
| Al-BP-Naph-273 K | 3.7128 | 0.5688 | 0.8708 | 0.999 |
| Al-BP-Naph-293 K | 6.2964 | 0.2758 | 0.9646 | 0.999 |

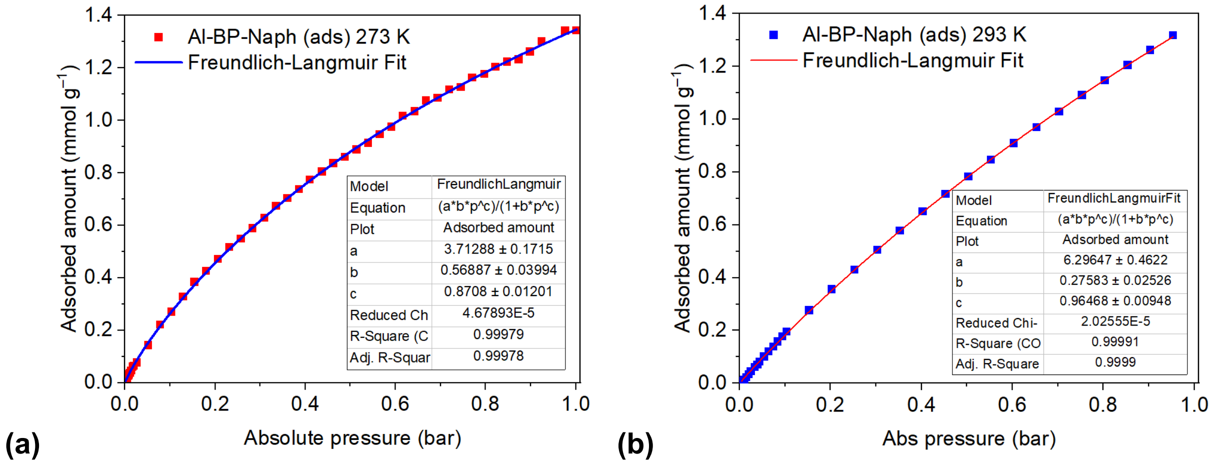


**Figure S14**. Adsorption of $CO_2$ at (**a**): 273 K and (**b**): 293 K, with the subsequent parameters of the Freundlich-Langmuir fit.

**Table S3**. Literature comparison between the $CO_2$ uptake and $CO_2$ heat of adsorption near zero coverage of Al-BP-Naph and other biphenyl-based MOFs.

| Sample | $CO_2$ uptake | | $CO_2$ $Q_{st}^0$ | Ref |
|---|---|---|---|---|
| | 293 K ($cm^3$ $g^{-1}$) | 273 K ($cm^3$ $g^{-1}$) | (kJ $mol^{-1}$) | |
| Al-BP-Naph | 29.4 | 30.11 | 26 | This work |
| DUT-5 | 36.2[(a)] | 48.6 | -[(b)] | 5 |
| COMOC-2 | 19.0 | 30.0 | 30 | 6 |
| UiO-67($NH_2$)$_2$ | -[(b)] | -[(b)] | 25 | 7 |
| MFM-300($Ga_2$) | 52.6 | 56.2 | 34 | 8 |
| MFM-300($Ga_{1.87}$ $Fe_{0.13}$) | 57.5 | 85.6 | 31 | 8 |
| UiO-67(1:2) | 27.0 | 57.3 | 43 | 9 |
| UiO-67-I(50) | 45.0 | 86.5 | 51 | 9 |
| BUT-10 | 50.6[(a)] | -[(b)] | 22 | 10 |
| BUT-11 | 53.5[(a)] | -[(b)] | 26 | 10 |
| UiO-67 | 22.9[(a)] | -[(b)] | 17 | 10 |

[(a)]This measurement is done at 298 K.
[(b)] Not mentioned

## Section S8. Photoluminescent properties

Selectivity: The photoluminescent (PL) properties of Al-BP-Naph in dimethylformamide (DMF) emulsion were investigated at room temperature. Metal ion quenching properties towards Al-BP-Naph were studied by introducing 3 mL of a 1 g $L^{-1}$ suspension of Al-BP-Naph into the cuvette and then adding 100 μL of a 0.1 mol $L^{-1}$ metal nitrate solution $M(NO_3)_x$ (M = $Co^{2+}$, $Cu^{2+}$, $Mg^{2+}$, $Ni^{2+}$, $Cd^{2+}$, $Fe^{3+}$, $Ni^{3+}$, $Pb^{2+}$, $Zn^{2+}$, $Mn^{2+}$, $Li^{+}$, $Ca^{2+}$, $Ag^{+}$ and $Cr^{3+}$).

Sensitivity for LOD: The PL properties of Al-BP-Naph containing various concentration $Fe^{3+}$ DMF solutions were also investigated using 3 mL of a 0.1 g $L^{-1}$ MOF suspension and a 0.001 mol $L^{-1}$ iron nitrate solution.

**Table S4**. Summary of the emission intensities from Figure 7a used to calculate the limit of detection (LOD).

| Added volume of $Fe^{3+}$ solution[(a)] (μL) | **Intensity I** (Cps) | Total volume (mL) | Final $Fe^{3+}$ concentration (mol $L^{-1}$) | $(I_0/I)-1$ |
|---|---|---|---|---|
| Blank 1 | 119106 | 3 | 0 | - |
| Blank 2 | 118903 | 3 | 0 | - |
| Blank 3 | 117074 | 3 | 0 | - |
| Blank 4 | 120194 | 3 | 0 | - |
| Blank 5 | 119900 | 3 | 0 | - |
| Blank 6 | 116524 | 3 | 0 | - |
| Blank 7 | 118306 | 3 | 0 | - |
| Blank 8 | 118597 | 3 | 0 | - |
| Blank 9 | 119048 | 3 | 0 | - |
| Blank 10 | 121846 | 3 | 0 | - |
| Blank 11 | 120039 | 3 | 0 | - |
| Blank 12 | 118425 | 3 | 0 | - |
| Blank 13 | 116698 | 3 | 0 | - |
| 20 | 115271 | 3.020 | $6.623\times10^{-6}$ | 0.030788 |
| 30 | 112377 | 3.030 | $9.901\times10^{-6}$ | 0.057334 |
| 40 | 106763 | 3.040 | $1.316\times10^{-5}$ | 0.112932 |
| 50 | 105437 | 3.050 | $1.639\times10^{-5}$ | 0.126929 |
| 60 | 102261 | 3.060 | $1.961\times10^{-5}$ | 0.161929 |
| 70 | 100416 | 3.070 | $2.280\times10^{-5}$ | 0.183278 |
| 80 | 98738 | 3.080 | $2.597\times10^{-5}$ | 0.203387 |
| 90 | 95949 | 3.090 | $2.912\times10^{-5}$ | 0.238366 |
| 100 | 93932 | 3.100 | $3.226\times10^{-5}$ | 0.264958 |

[(a)] Concentration of $Fe^{3+}$ solution: 0.001 mol $L^{-1}$.

Mean blank ($I_0$) = 118820

$\sigma_{Blank} = \sqrt{\frac{\sum(I-I_0)^2}{(N-1)}}$ = 1503 (N = 13)

**Limit of detection (LOD) and limit of quantification (LOQ):**

σ standard deviation of the blank measurements and k is the slope of fitting line of fluorescence intensity versus analyte ion concentration (Figure 7c in the main text)

$LOD = 3\sigma/k = 3 \times 1503/ (801913000\ L\ mol^{-1}) = 5.6\ \mu mol\ L^{-1}$

$LOQ = 10\sigma/k = 10 \times 1503/ (801913000\ L\ mol^{-1}) = 18.8\ \mu mol\ L^{-1}$

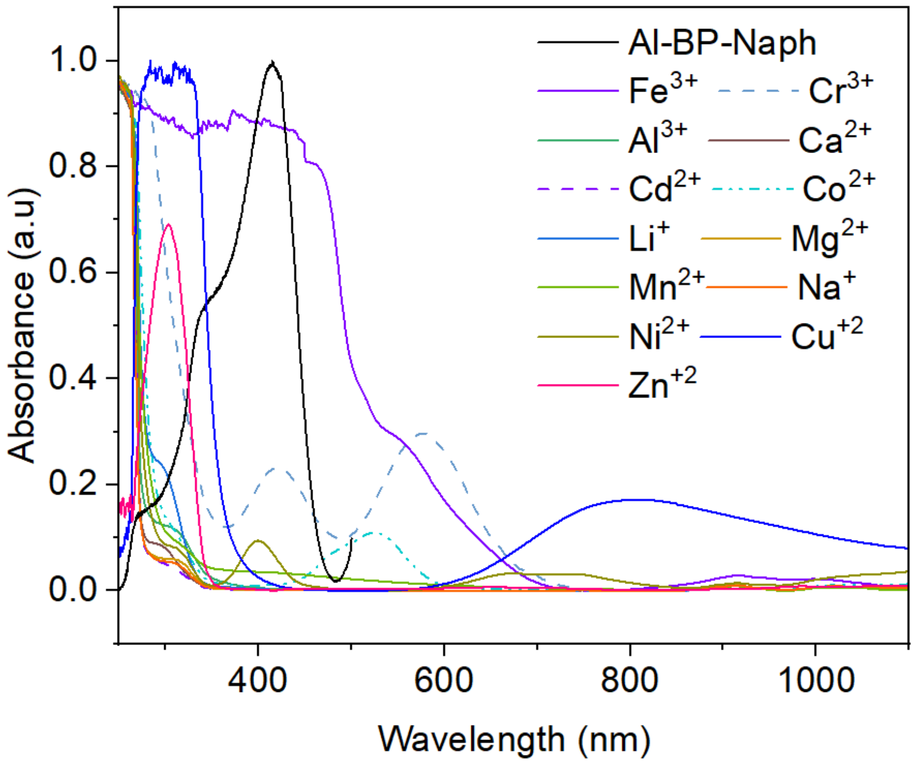


**Figure S15**. UV/Vis absorbance spectra of the different metal nitrate salts (0.1 mol $L^{-1}$) and the excitation spectrum of the Al-BP-Naph suspension in DMF (c = 1 g $L^{-1}$).

$Co^{2+}$ has its absorption maximum at 525 nm with a range from 470 to 550 nm which is outside the main absorption of the Al-BP-Naph suspension.
Instead $Cr^{3+}$ has an absorption at 415 nm with a range from 370 to 471 nm which explains why $Cr^{3+}$ also slightly decreases the intensity of the MOF.
Also, $Zn^{2+}$has an absorption maximum at 370 nm with a range between 265 to 340 nm that overlaps with the high energy shoulder of the excitation spectrum of the MOF.
Thus, $Cr^{3+}$ and $Zn^{2+}$ can also compete with the MOF in absorbing electromagnetic energy and this translates to decreased emission intensity in Figure 6a and better represented in Figure 6b.
$Cu^{2+}$ has its absorption maxima at 303 nm and 800 nm with ranges from 270 to 330 nm and 620 to 1000 nm. The first maximum at 300 nm overlaps only very slightly with the high-energy shoulder of the excitation spectrum of the Al-BP-Naph suspension but is otherwise largely outside the main absorption of the Al-BP-Naph suspension.

## Section S9. References


1. Cseri, L.; Hardian, R.; Anan, S.; Vovusha, H.; Schwingenschlögl, U.; Budd, P. M.; Sada, K.; Kokado, K.; Szekely, G. Bridging the interfacial gap in mixed-matrix membranes by nature-inspired design: precise molecular sieving with polymer-grafted metal–organic frameworks. *J. Mater. Chem. A* **2021**, *9 (42)*, 23793-23801. DOI: 10.1039/D1TA06205K.

2. Petříček, V.; Dušek, M.; Palatinus, L. Crystallographic Computing System JANA2006: General features. *Z. Kristallogr.* **2014**, *229* (5), 345–352.DOI: 10.1515/zkri-2014-1737

3. Liu, Y.-Y.; Couck, S.; Vandichel, M.; Grzywa, M.; Leus, K.; Biswas, S.; Volkmer, D.; Gascon, J.; Kapteijn, F.; Denayer, J. F. M.; et al. New VIV-Based Metal–Organic Framework Having Framework Flexibility and High $CO_2$ Adsorption Capacity. *Inorg. Chem*. **2013**, *52 (1)*, 113-120. DOI: 10.1021/ic301338a.

4. Nuhnen, A.; Janiak, C. A practical guide to calculate the isosteric heat/enthalpy of adsorption via adsorption isotherms in metal–organic frameworks, MOFs. *Dalton Trans*. **2020**, *49 (30)*, 10295-10307. DOI: 10.1039/D0DT01784A.

5. Raja, D. S.; Chang, I.-H.; Jiang, Y.-C.; Chen, H.-T.; Lin, C.-H. Enhanced Gas Sorption Properties of a New Sulfone Functionalized Aluminum Metal-Organic Framework: Synthesis, Characterization, and DFT Studies. *Microporous Mesoporous Mater.* **2015**, *216*, 20–26. https://doi.org/10.1016/j.micromeso.2015.02.023.

6. Wang, G.; Leus, K.; Couck, S.; Tack, P.; Depauw, H.; Liu, Y.-Y.; Vincze, L.; Denayer, J. F. M.; Van Der Voort, P. Enhanced gas sorption and breathing properties of the new sulfone functionalized COMOC-2 metal organic framework. *Dalton Trans.* **2016**, *45 (23)*, 9485-9491, DOI: 10.1039/C6DT01355D.

7. Ko, N.; Hong, J.; Sung, S.; Cordova, K. E.; Park, H. J.; Yang, J. K.; Kim, J. A significant enhancement of water vapour uptake at low pressure by amine-functionalization of UiO-67. *Dalton Trans.* **2015**, *44 (5)*, 2047-2051, 10.1039/C4DT02582B. DOI: 10.1039/C4DT02582B

8. Krap, C. P.; Newby, R.; Dhakshinamoorthy, A.; García, H.; Cebula, I.; Easun, T. L.; Savage, M.; Eyley, J. E.; Gao, S.; Blake, A. J.; et al. Enhancement of $CO_2$ Adsorption and Catalytic Properties by Fe-Doping of [$Ga_2$(OH)2(L)] ($H_4$L = Biphenyl-3,3′,5,5′-tetracarboxylic Acid), MFM-300($Ga_2$). *Inorg. Chem.* **2016**, *55 (3)*, 1076-1088. DOI: 10.1021/acs.inorgchem.5b02108

9. Wang, B.; Zeng, J.; He, H. Enhancing $CO_2$ adsorption capacity and selectivity of UiO-67 through external ligand modification. *Sep. Purif. Technol.* **2025**, *354*, 128651. DOI: https://doi.org/10.1016/j.seppur.2024.128651.

10. Wang, B.; Huang, H.; Lv, X.-L.; Xie, Y.; Li, M.; Li, J.-R. Tuning $CO_2$ Selective Adsorption over $N_2$ and $CH_4$ in UiO-67 Analogues through Ligand Functionalization. *Inorg. Chem.* **2014**, *53* (17), 9254-9259. DOI: 10.1021/ic5013473.

11. Xu, H.; Dong, Y.; Wu, Y.; Ren, W.; Zhao, T.; Wang, S.; Gao, J. An -OH group functionalized MOF for ratiometric $Fe^{3+}$ sensing. *J. Solid State Chem*. **2018**, *258*, 441-446. DOI: https://doi.org/10.1016/j.jssc.2017.11.013.
